# Operations research in global health: a scoping review with a focus on the themes of health equity and impact

**DOI:** 10.1186/s12961-017-0187-7

**Published:** 2017-04-18

**Authors:** Beverly D. Bradley, Tiffany Jung, Ananya Tandon-Verma, Bassem Khoury, Timothy C. Y. Chan, Yu-Ling Cheng

**Affiliations:** 1 Centre for Global Engineering, University of Toronto, Toronto, ON Canada; 2 Department of Chemical Engineering and Applied Chemistry, University of Toronto, 200 College St, Toronto, ON M5S 3E5 Canada; 3 Department of Mechanical and Industrial Engineering, University of Toronto, Toronto, ON Canada; 4 Centre for Healthcare Engineering, University of Toronto, Toronto, ON Canada

**Keywords:** Operations research, Modelling, Policy, Decision-making, Global health, Health systems, Health equity, Developing countries, Low-resource settings

## Abstract

**Background:**

Operations research (OR) is a discipline that uses advanced analytical methods (e.g. simulation, optimisation, decision analysis) to better understand complex systems and aid in decision-making.

**Summary:**

Herein, we present a scoping review of the use of OR to analyse issues in global health, with an emphasis on health equity and research impact. A systematic search of five databases was designed to identify relevant published literature. A global overview of 1099 studies highlights the geographic distribution of OR and common OR methods used. From this collection of literature, a narrative description of the use of OR across four main application areas of global health – health systems and operations, clinical medicine, public health and health innovation – is also presented. The theme of health equity is then explored in detail through a subset of 44 studies. Health equity is a critical element of global health that cuts across all four application areas, and is an issue particularly amenable to analysis through OR. Finally, we present seven select cases of OR analyses that have been implemented or have influenced decision-making in global health policy or practice. Based on these cases, we identify three key drivers for success in bridging the gap between OR and global health policy, namely international collaboration with stakeholders, use of contextually appropriate data, and varied communication outlets for research findings. Such cases, however, represent a very small proportion of the literature found.

**Conclusion:**

Poor availability of representative and quality data, and a lack of collaboration between those who develop OR models and stakeholders in the contexts where OR analyses are intended to serve, were found to be common challenges for effective OR modelling in global health.

**Electronic supplementary material:**

The online version of this article (doi:10.1186/s12961-017-0187-7) contains supplementary material, which is available to authorized users.

## Background

‘Global health’ broadly refers to “*an area for study, research, and practice that places a priority on improving health and achieving equity in health for all people worldwide*” [1]. From population-based prevention to individual-level clinical care, global health encompasses health issues and solutions that transcend borders, and involves a collaborative and interdisciplinary effort [1]. The goal of achieving equity in health, namely the absence of systematic disparities in health or in the major social determinants of health between groups with different levels of underlying social advantage/disadvantage [2], has become a particularly important part of the post-2015 development agenda [3–6]. Globally, major progress has been made towards certain Millennium Development Goals and targets; however, many low- and middle-income countries (LMICs), especially in sub-Saharan Africa and Asia, continue to experience high health inequities, both within and between countries [3, 4]. Further, these countries carry most of the world’s burden of morbidity and mortality; for example, more than 99% of under-5 child deaths in 2010 occurred in LMICs, and although mortality rates fell in most monitored countries, 15 countries experienced increases in the absolute number of deaths, with 12 of these countries being in sub-Saharan Africa [7].

Operations (or operational) research (OR) is a discipline that uses advanced analytical methods to better understand complex systems and aid in decision-making [8, 9]. OR uses a wide range of problem-solving techniques and computational methods, including computer simulation, mathematical optimisation, statistics and decision analyses, to help improve the operations of organisations. With its orientation towards improving efficiency, cost-effectiveness and decision-making, OR is particularly useful for analysing complex global health issues – especially in settings where the burden of disease is high but health systems are weak and resources limited. Despite the growing use of OR in global health, it is unknown how much of an impact OR is having in this important area as publications rarely discuss whether their findings were implemented or were influential in policy- or decision-making [10, 11].

The objective of this scoping review is to examine the extent, range and nature of operations research activity in global health, specifically within healthcare settings, health services delivery, and population health in LMICs. Our goal is to highlight the breadth of healthcare applications of OR in global health, both geographically and across application areas, and – through select case studies – discuss the impact such studies can have on improving healthcare and healthcare equity for communities and populations globally. We aim to encourage OR researchers and global health practitioners alike to continue to apply OR in global health, particularly in areas where OR-based studies may currently be lacking, and to consider sharing the impact of OR work more broadly so that others can learn from challenges and successes.

It should be noted that, in the context of global health, the term ‘operations research’ is sometimes synonymous with implementation research [12] and is used broadly to encompass cross-sectional, case-control, retrospective or prospective cohort analyses [13–15], as well as qualitative research methods [12, 16], all of which are valuable in studying the effectiveness of health services and programs within the day-to-day operating environments of routine practice. In this review, however, we focus on studies where analytical methods or modelling are used to explore health research questions with an orientation towards decision-making or the consideration of ‘what-if’ scenarios – in other words, modelling studies that are prescriptive in their recommendations.

The modelling realm of OR is of particular interest because it can help address global health questions not easily answered with other methods. For example, OR is beneficial in situations where conducting a real-world study might be considered impossible, impractical, too costly or unethical, such as when choosing between implementing policy ‘A’ or policy ‘B’, when controlled trials to compare a wide variety of available options would be unreasonable, when the disease or illness of interest takes years or decades to progress and the process of evaluating long-term outcomes would be long and expensive, or when simulating virtual cohorts of patients allows researchers to explore questions without ethical consequences. OR is also useful for framing complex financial evaluations, for example, determining the most cost-effective intervention among many options, establishing the optimal way to allocate a limited budget across multiple competing needs, or deciding whether a new intervention (e.g. a vaccine) can be implemented sustainably with limited funding. In LMICs, such OR analyses, which help narrow down the number of possible options or help inform where to focus efforts for more targeted studies, are even more important due to limited resources.

While OR in healthcare in the developed world has been extensively studied in recent years [17–22], the latest review of OR in healthcare in developing countries was published in 1993 [10]. A few recent review papers and bibliographies have explored the use of OR in developing countries; however, these did not specifically focus on healthcare and included several other sectors such as agriculture, energy and transport [11, 23–25]. Others have reviewed the use of OR within a very narrow area of global health (e.g. infectious diseases, particularly HIV) [26–30]. Several survey papers and special issues of journals have recently focused on the use of OR to address global health or humanitarian issues, but these were not based on systematic reviews of the literature [16, 31–33]. Given that the existing literature on this topic is sporadic, not comprehensive in the search strategy, and lacks depth in the analysis of thematic areas, we have chosen a broad scoping review approach. With this approach, we aim to build upon previous work by providing a systematic and comprehensive landscape overview of the use of OR in global health with a more rigorous analytic framework than has been previously performed.

The results of this scoping review are presented in four main sections. First, we present a global overview of the literature, which includes the distribution of OR studies across countries of different income classifications, over time, and across different methodological approaches. Then, we explore the use of OR in four global health application areas with concrete examples in each category. In this review, we consider the four main application areas of global health to be (1) health systems and operations, (2) clinical medicine, (3) public health, and (4) health innovations – from the local to global level. Next, health equity, which is integral to the concept of global health and transcends all four application areas, is explored as a separate overarching theme using a subset of included studies. Health equity is not only a topic of growing interest globally, but is compelling to explore through an OR lens. For example, when health equity is operationalised and quantified using meaningful and measurable criteria [2], OR methods can be used to analytically find solutions that improve or maximise equity. To our knowledge, the use of OR to analyse issues of health equity has not yet been explored through a systematic review. Finally, by way of selected cases, we present a discussion on implementation and impact, i.e. how OR has influenced real health policy change or aided in decision-making by stakeholders. We highlight common factors among these studies that likely helped contribute to their effective translation into policy or practice, and discuss barriers and challenges to bridging the gap between OR and health policy. We conclude the paper with a discussion of key insights and implications of this review.

## Methods

We followed the scoping review framework set out by Arksey and O’Malley [34] and by others who have proposed refinements to this approach [35–37]. Specifically, we followed these five stages:^1^ (1) identifying the research question; (2) identifying relevant studies; (3) study selection; (4) charting the data; and (5) collating, summarising and reporting results.

### Stage 1: Identifying the research question

This scoping review seeks to identify the extent, range and nature of OR in global health (i.e. geographical, over time, methodological and across application areas), with an in-depth exploration of literature addressing questions of health equity and literature having made a specific impact in decision-making or policy change.

### Stage 2: Identifying relevant studies

The databases HealthStar (a subset of Ovid Medline focused on health systems research), Scopus, Web of Science, Inspec and Compendex were chosen to capture literature from multidisciplinary sources across health research and engineering. Individualised search strategies were designed for each database.^2^ We searched titles, abstracts and keywords for combinations of search terms in the following categories: OR modelling and methodologies; healthcare settings, health services delivery, and population health; LMICs and regions (e.g. sub-Saharan Africa, South East Asia, etc.), including specific country names in these income categories; and policy- and decision-making. The search strategy including all search terms for the Web of Science database is provided in Box 1 as an illustration, and all others are provided in Additional file 1: Tables S1 to S4. Only papers published in the year 2000 and later were included. This search strategy was refined and validated by ensuring the search captured a set of 15 ideal target papers [38–52] known to the authors. Librarians specialising in both engineering and health sciences literature were consulted when designing the search strategy. Search results were downloaded in August 2014. We also hand-searched 19 review papers and special issue articles [11, 13, 16, 17, 19, 23–33, 53–55] for additional references.

**Box 1** Example search strategy for Web of Science database

**Table:** 

Database: Web of Science (WOS)
Strategy: Keyword search. “Topic search” (TS) was used, which searches all words in article titles, abstracts, author keywords, and “KeyWords Plus” fields. Sub-search categories: (a) model types, (b) geographic focus, (c) health, and (d) decision-/policymaking. Terms within categories were combined with “or”, and categories were combined with “and”. All LMIC names individually listed since country names are not controlled vocabulary in WOS. Results pre-2000 and unrelated WOS categories were excluded.
Sub-search categories: *(a) Model types* TS=(“operation* research”) OR TS=(model* NEAR/5 (mathematical or queu* or inventory or scheduling or demand or forecast* or comput* or network or stochastic or decision* or delivery or simulation or optimi?ation or non-linear or nonlinear or linear or Markov or cost-effectiveness or agent-based)) OR TS=(optimi?ation$ NEAR/5 (mathematical or nonlinear or non-linear or linear or network or discrete or multicriteria or multi-criteria or stochastic or problem or minimi?ation or maximi?ation or location or allocation)) OR TS=(simulation NEAR/3 (comput* or discrete or agent-based or system$))
AND
*(b) Geographic focus* TS=(“developing countr*” OR “low-income countr*” OR “middle-income countr*” OR “developing world” OR “developing nation*” OR “low-resource setting*” OR “resource-constrained setting*” OR “resource-poor setting*” OR “limited-resource setting*” OR “resource-limited setting*” OR “under-developed countr*” OR “least-developed countr*” OR “less-developed countr*” OR LMIC* OR Africa* OR (Asia* NEAR/2 south) OR (Asia* NEAR/2 east) OR “latin America*” OR “central America*” OR “south America*” OR Caribbean OR “middle east”) OR *[all low- and middle-income country names listed out]*
AND
*(c) Health* TS=(health* or medical or hospital or clinic* or treatment) OR AD=(health or hlth)
AND
*(d) Decision-/policy-making* TS=(polic* or decision-mak* or decision-support or decision-process or decision-aid* or implement* or impact) OR AD=(policy)

**Fig. 1:**
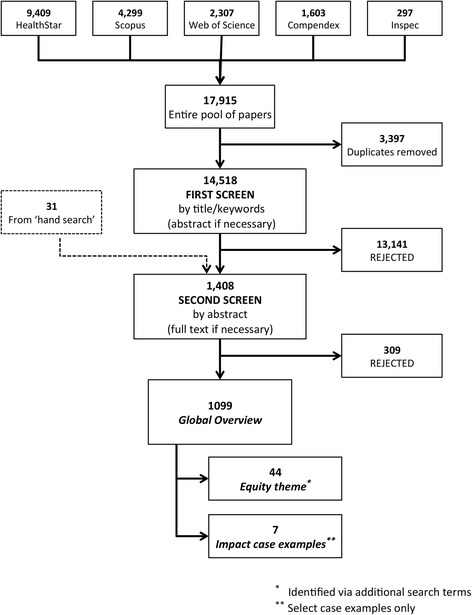
Systematic search results and screening process

Search results from each database were combined and duplicates were removed. An initial screen of the remaining 14,518 references eliminated studies that were clearly not relevant to this review. This initial screen was largely based on title and keywords; if additional information was required to judge relevance, the abstract was consulted. A large proportion of papers rejected at this stage fell into one of two categories, either (1) field studies and implementation research that did not have a modelling element, or (2) health-related modelling studies that were purely explanatory or descriptive in nature and did not have an orientation towards policy- or decision-making. There were 1408 abstracts remaining after this initial screening, including 31 articles from hand-searching review papers (Fig. 1).

### Stage 3: Study selection

A second screen was conducted whereby a more rigorous set of inclusion criteria was applied to identify the final set of papers for the review. Two co-authors independently reviewed each abstract against the following four key inclusion criteria: (1) the study clearly used methodologies common to the field of OR; (2) the problem or research question was of an OR nature; (3) the study was related to a healthcare or public health issue; and (4) the geographic focus was on LMICs and/or regions (Box 2). For each main criterion, at least one of the sub-points had to be true in order for a paper to be included in the review. Papers for which both reviewers were in agreement were automatically included. Discrepancies were resolved through discussion (with a third reviewer if necessary), or by downloading and reading the full-text. Co-authors met periodically during this stage to discuss any uncertainties related to study selection and ensure consistency in applying the criteria. After applying the inclusion criteria, 1099 papers remained – these comprised the set of studies for our global overview.

**Box 2** Inclusion criteria for second screen of review process

**Table:** 

1) An operations research (OR) technique is used	
↑ or ↓	□ Simulation model (e.g. discrete event, agent-based, etc.) □ Optimisation model (e.g. linear and non-linear programming, goal programming, location-allocation models) □ Decision analysis, decision tree models □ Stochastic, compartmental, state transition models (e.g. Markov) □ General mathematical or probabilistic models of disease progression and/or transmission will be considered *if* Criteria 2 is met
and	
2) An OR problem is explored^a^	
↑ or ↓	□ Several competing interventions and/or policy options are modelled/simulated/compared to propose the best/optimal strategy (incl. comparing current status quo vs. a new option) □ The cost-effectiveness of a treatment or intervention is explored through hypothetical cohorts of patients or through decision-analysis techniques to estimate costs per person □ Outcomes are modelled for different treatment or therapy scenarios/options □ Issues of logistics, supply chain, distribution, scheduling are explored; including studies that highlight operational inefficiencies or poor performance □ ‘What if’ scenarios are tested, e.g. what if anti-retroviral therapy coverage was increased?
and	
3) The OR problem has a healthcare delivery or public health focus	
↑ or ↓	□ Health services delivery (hospital/clinical services, primary care, treatment or diagnostic options, health technology management or integration, etc.) □ Public or population health (vaccination policy, mass screening for health conditions, transmission prevention and/or reduction, etc.)
and	
4) The study focuses on a low- or middle-income setting	
↑ or ↓	□ Country-focused: a look-up table was provided to co-authors to determine whether a country was low income, lower-middle income or upper-middle income^b^ □ Regional: Africa, sub-Saharan Africa, South East Asia, Latin America, South America, etc.

^a^This list does not cover the full extent of OR-type problems, but these criteria describe the types of OR problems of interest for this review^b^According to World Bank classifications as of July 1, 2014. Low-income economies are defined as those with a gross national income (GNI) per capita of $1045 or less in 2013; middle-income economies are those with a GNI per capita of more than $1045 but less than $12,746; lower-middle- and upper-middle-income economies are separated at a GNI per capita of $4125

To identify papers among the 1099 studies in the global overview that explore the specific theme of health equity, we searched for the following keywords within titles, abstracts and author addresses: (in)equit*, (in)equalit*, *pro-poor*, *poorest*, *socio-economic*, *marginalized*, stigm*, quintile*, disparit* and *gender*. Two co-authors assessed each abstract and collaboratively decided if they addressed an issue aligned with the definition of health equity as described by Braveman and Gruskin [2]. Full-texts for health equity-themed papers were downloaded and read.

Identifying studies for the impact theme was less straightforward. As noted by others who have reviewed OR in global health or LMICs [10, 11, 23, 24], many OR studies are published before any evidence of having influenced policy- or decision-making has been demonstrated. Thus, it would be misleading to assess the impact of OR studies based solely on a review of published literature. We therefore took the approach of providing select case examples of studies where impact was described in the publication in order to gain insight from their experiences, with the caveat that additional OR studies have likely had an impact on improving global health. For our purposes, ‘impact’ implies that study results meaningfully informed a policy decision, or that model recommendations were implemented in a real-world situation. Full-texts for impact-themed papers were downloaded and read.

### Stage 4: Charting the data

‘Charting’ is “*a technique for synthesising and interpreting qualitative data by sifting, charting and sorting material according to key issues and themes*” [34]. For each of the studies included, the following items of information were charted by one co-author and cross-checked by another: (1) country or region of focus; (2) income classification of that country^3^ – low income, lower-middle income, or upper-middle income; (3) OR methodology or type of OR model developed/used for the analysis; (4) health issue studied (HIV/AIDS, malaria, childhood pneumonia, etc.); (5) application area of global health – Health Systems and Operations, Clinical Medicine, Public Health, or Health Innovation; and (6) level at which the study was targeted – local, national, regional, global or general. These characteristics were gathered from the abstract, but in cases where this information was not clearly stated, the full text was consulted.

The Health Systems and Operations category refers to studies that looked at the logistics related to the provision of services, the allocation of resources or the operations of health facilities. Clinical Medicine was distinguished from public health in that these studies focused primarily on disease diagnosis, treatment or care for the individual patient (e.g. treatment regimens, case management, etc.), whereas Public Health studies emphasised disease prevention and health promotion at the community or population level (e.g. vaccination policy, mass screening, etc.). The Health Innovation category was reserved for studies that explored healthcare innovations or technologies in the pre-implementation stages of development (e.g. vaccines still in early clinical trials and not yet accepted for widespread use, hypothetical future discoveries – in diagnostic, treatment or vaccine technologies). The study’s target level refers to the level at which the model recommendations would be or were intended to be implemented. ‘Regional’ refers to global regions (e.g. sub-Saharan Africa, South-East Asia) and not sub-national regions. The ‘general’ category was reserved for those studies that considered ‘low-resource settings’ as the target in a very general sense or where the level of intended implementation was not clear.

For the most part, studies were easily categorised; however, a small fraction fell into grey areas. Where there was overlap, a determination was made based on what was deemed to be the dominant category. For example, studies that were based on a local setting but were intended to inform national policy- or decision-making were counted towards the national category because proposed changes would be made at the national level. Similarly, some studies bridged clinical and public health (e.g. screen and treat programs). We considered any study with broad public health goals, regardless of whether treatment was included, as public health.

### Stage 5: Collating, summarising and reporting results

Based on Arksey and O’Malley [34], we present our findings in two ways. First, through basic numerical analysis of the extent, nature and distribution of studies across various dimensions (i.e. global overview, application areas), and second by organising a subset of the literature thematically (i.e. for the themes of health equity and impact).

## Results

### Global overview

In this section, we present a global overview of the 1099 studies that met the inclusion criteria, including a breakdown of OR studies according to country income classifications, geographic regions, year of publication and methodology.

**Fig. 2:**
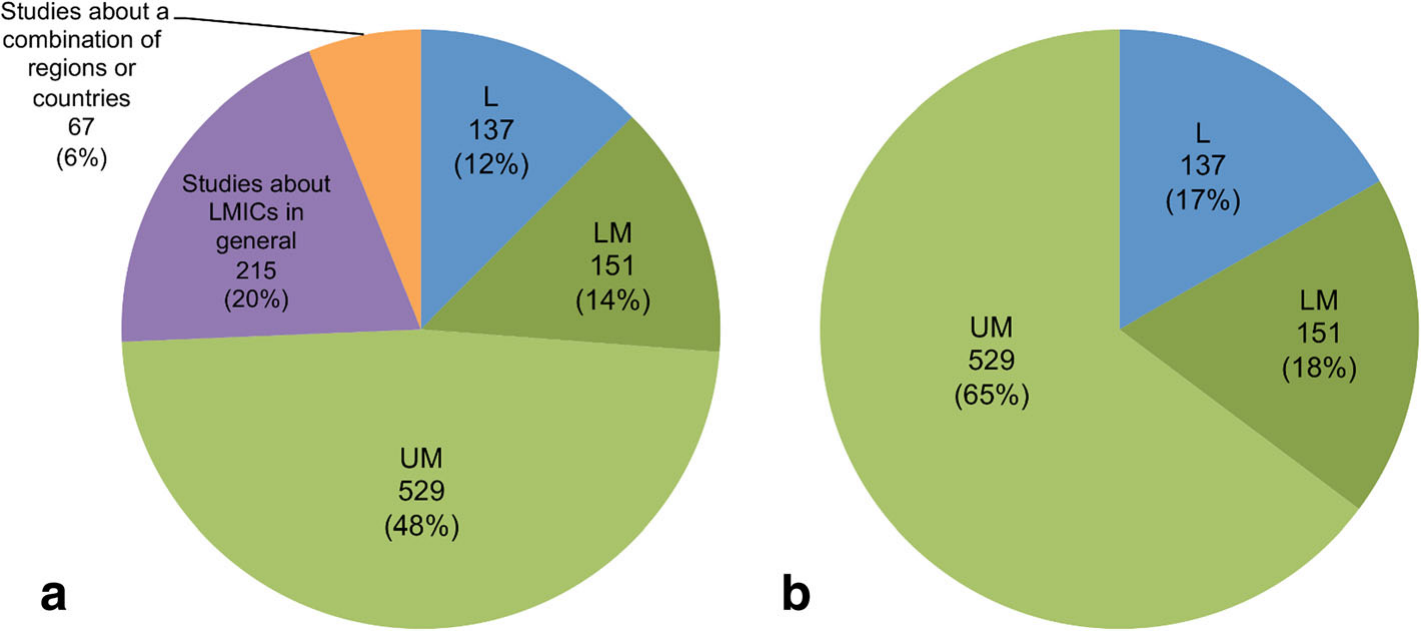
Breakdown of operations research studies according to World Bank income classification of the country of focus – low-income (L), lower-middle-income (LM) or upper-middle-income (UM) – for (**a**) all studies (*n* = 1099) including studies about low- and middle-income countries in general or some combination of regions and/or L-, LM- and UM-income countries; and (**b**) studies focused on a single country only (*n* = 817)

Figure 2 shows the breakdown of OR studies by country income level. The majority of studies (74%) were focused on a specific low, lower-middle, or upper-middle income country; however, several studies (20%) were targeted towards LMICs broadly, and a small proportion (6%) looked specifically at a grouping of countries or regions that spanned several income categories (Fig. 2a). Among the 817 studies that had a single-country focus (Fig. 2b), low-income countries made up 17% whereas middle-income countries (lower-middle and upper-middle) made up 83%, with the majority being in the upper-middle income category. Using number of countries and population as benchmarks,^4^ our findings suggest that lower-middle-income countries are under-represented in the literature, upper-middle-income countries are over-represented, and the representation of low-income countries is roughly proportional to these benchmarks. Lower-middle-income countries make up about 34% of all LMICs and 44% of the LMIC population but only 18% of the literature, while upper-middle-income countries make up 40% of all LMICs and 41% of the LMIC population but 65% of the literature. For comparison, low-income countries, which make up 17% of the literature, represent approximately 26% of all LMICs globally and 15% of the LMIC population.

**Fig. 3:**
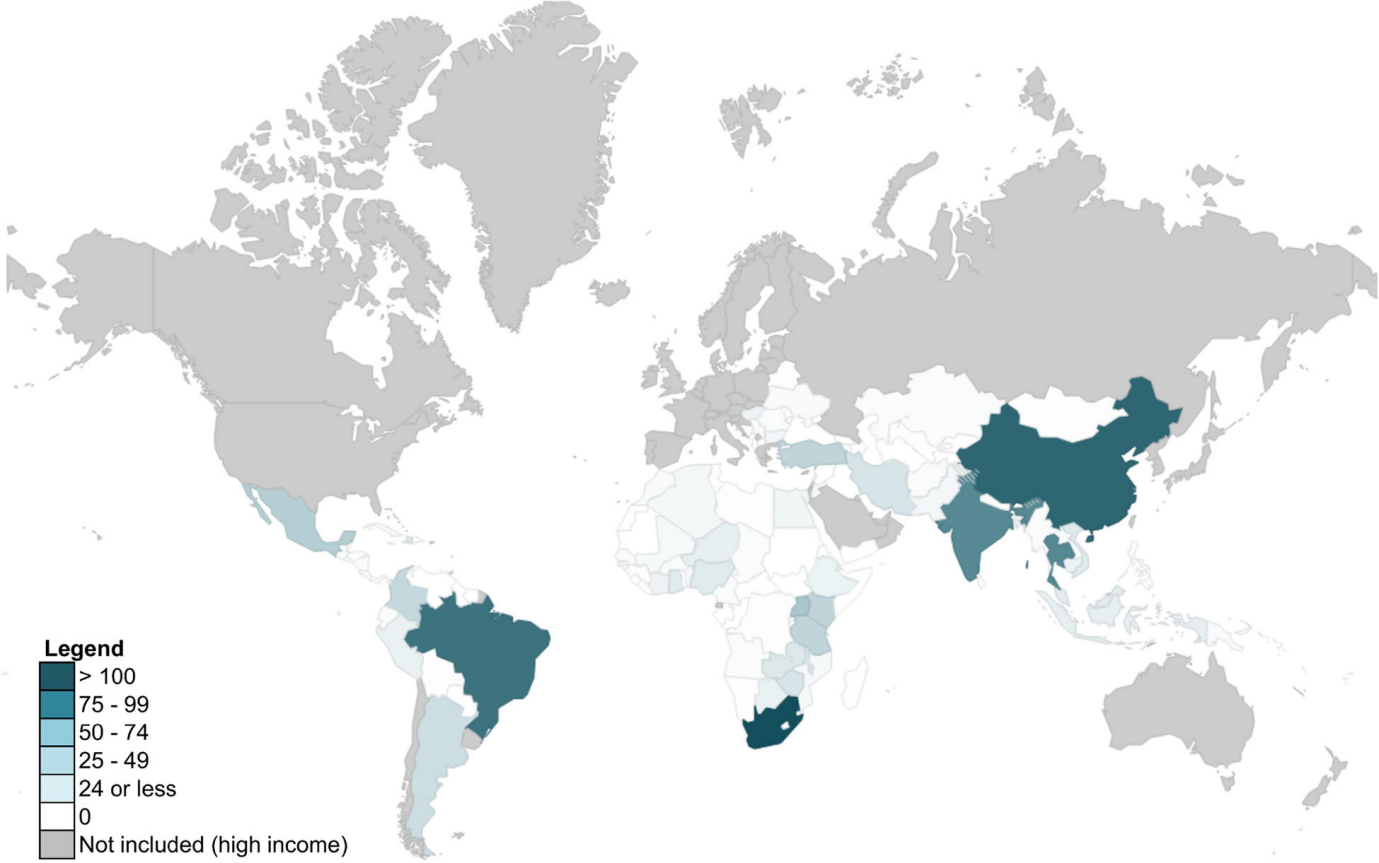
Number of operations research studies by country. Note that only studies that focused on a single country (*n* = 817) or multiple specific countries (*n* = 55) are represented in this figure. Studies that considered multiple countries are counted once for each country represented

Figure 3 provides a more detailed geographical view of the distribution of OR studies across the developing world. Almost 40% of the literature reviewed was focused on just six LMICs. China, Brazil and South Africa were the most frequently studied, and collectively accounted for 25.4% of the studies reviewed. India, Mexico and Thailand accounted for 14.5%; all were classified as upper-middle-income countries, except India, which was a lower-middle-income country. These countries represent just 4.4% of all LMICs, but account for about 52% of the total LMIC population. The low-income country most studied in the OR literature was Uganda, with 26 studies. More papers were focused on Asia and South America than sub-Saharan Africa (excluding South Africa). Approximately 50 LMICs were not studied in any of the global health OR publications identified; these countries account for approximately 5% of the total LMIC population, or approximately 303 million people.

**Fig. 4:**
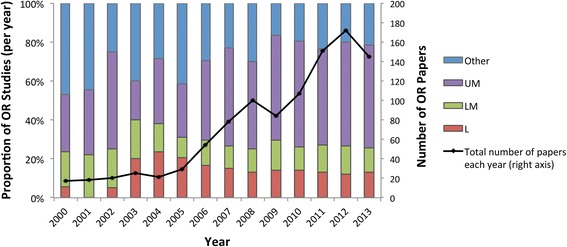
Proportion of operations research (OR) studies per year in different country income classifications (bars, left axis); low income (L), lower-middle income (LM), upper-middle income (UM) and Other (includes studies targeted at LMICs in general or some combination of L-, LM- and UM-income countries). Total number of OR papers per year also displayed (line, right axis). Note that 2014 was excluded as this review covers studies indexed up until August 2014 only

As Fig. 4 suggests, low- and lower-middle income countries have historically been less frequent targets for global health-related OR compared to upper-middle-income countries. Despite a steady increase in the absolute number of studies focused on low-income countries since 2000, the proportion of such studies relative to all global health-related OR has plateaued at approximately 14% since the year 2006. This figure also suggests a trend towards more country-specific analyses rather than studies that consider LMICs in general or groupings of countries (see Other category in Fig. 4). A possible explanation for the drop in number of papers for 2013 is the lag between when a paper is published versus when it has been indexed in databases. The year 2014 was not included in Fig. 4 since our review does not encompass the entire year.

**Fig. 5:**
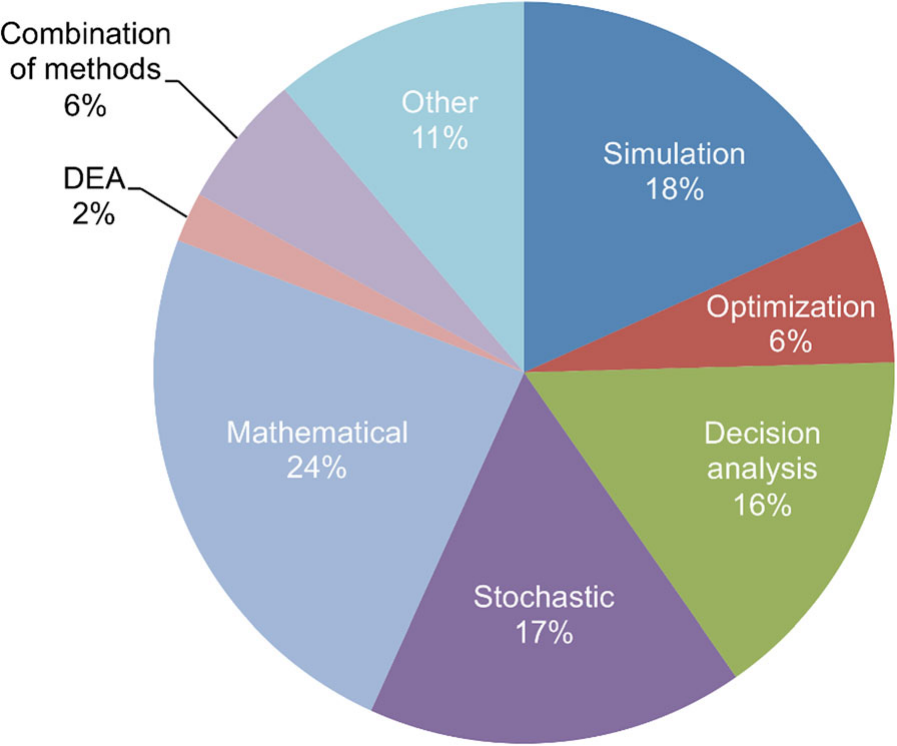
Breakdown of operations research methodologies. The Stochastic category includes Markov models (e.g. state-transition and decision process models) and Monte Carlo methods. The Mathematical category includes deterministic models, epidemiological compartmental models, and other analytical models described by governing equations. The Other category includes all remaining smaller categories including artificial neural networks, inventory models, spreadsheet models with no analytical formulation, etc. See Box 2 Criterion (1) for additional details about the methodologies included. *DEA* data envelopment analysis

A breakdown of OR studies according to methodology is shown in Fig. 5. A wide range of OR methods have been used to study global health issues, and no single method appears to be dominant. In the section that follows, examples of different methods are provided within the context of four application areas of global health.

### Global health application areas

**Fig. 6:**
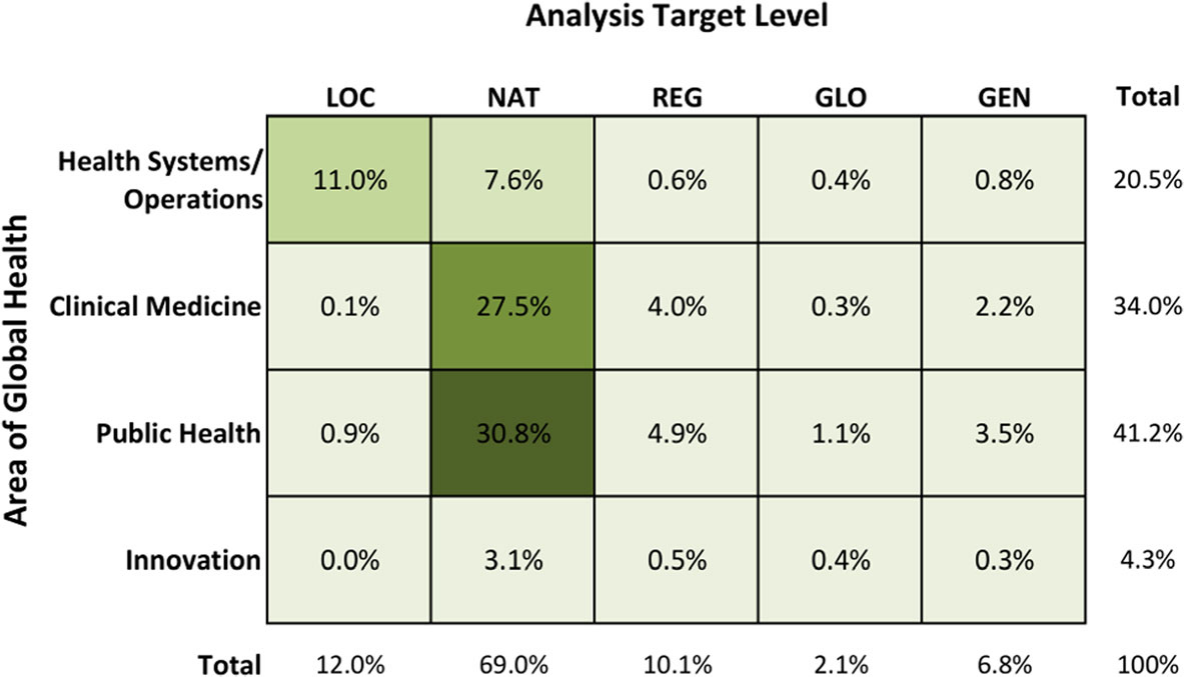
Percentage of operations research studies (*n* = 1099) by application area of global health and by analysis target level (local, national, regional, global or general)

In this section, we explore the volume and breadth of OR literature found across two dimensions of global health; the global health application area and the level at which the analysis was targeted (Fig. 6). These application areas were chosen because we felt they were broad enough to cover the full gamut of global health challenges. At the same time, studies within categories would carry a similar flavour in the types of problems studied. Other categorisations could also have been appropriate [10, 16]. Similarly, we felt it important to distinguish between different levels of focus as the types of problems, analytical approaches, and scale of implementation would be different across these levels. Detailed examples of OR studies in the four areas of global health are described in more detail in the sub-sections that follow.

The majority (58.3%) of OR literature explores clinical medicine and public health issues at a national level, with locally-focused health systems and operations studies being the next most frequently studied area (11.0%). Since policies affecting clinical medicine and public health are typically mandated by national ministries of health or implemented by national public health programs, it makes sense that the OR studies in these areas have been targeted at the national level. Although very helpful for exploring the impact of interventions on a macro scale or adding to discussions on global priority-setting, fewer studies were targeted at the regional or global level (12.2%) or towards LMICs in general (6.8%).

#### Health systems and operations

About 20% of the literature was related to health systems and operations, and most of these studies were focused on the local or national level. At a local level, common analyses included improving the day-to-day operations of health facilities (e.g. patient flow and wait times in health facilities [49, 56–60] and emergency departments [61–64], facilities layout planning [65, 66], inventory planning [67, 68], nurse rostering [69–75], and surgical scheduling [76–79]) and health services planning (e.g. location-allocation of emergency medical services [80–82] or new health facilities [83–86]). The majority of these types of problems were analysed using simulation (25.0%) or optimisation (35.0%), or a combination of both methods (3.3%). For example, discrete-event simulation (DES) was used to analyse patient wait times in health clinics in Zambia [49] and Colombia [59], and a combination DES-optimisation model was used to study ambulance positioning and response time for an urban city in Brazil [81].

At the national level, questions related to supply chains and logistics were often explored using methods such as agent-based simulation, DES and other types of microsimulation. For example, a series of recent studies used the HERMES (Highly Extensible Resource for Modeling Event-Driven Supply Chains) software to develop DES models of the vaccine supply chain in Niger and Thailand; researchers explored the impact on vaccine availability of introducing new vaccines into the supply chain [87, 88], changing vaccine vial size or replacing multi-dose vials with single-dose vials [46, 89], removing the regional level of distribution [90], and trade-offs between augmenting transport versus increasing cold storage capacity [47, 91].

The lack of adequate resources represents a major constraint for health systems in LMICs, thus the efficiency with which available resources are being used was another common theme. A small subset of OR literature (2%) used data envelopment analysis at both a local and national level to analyse the efficiency of health facilities and systems in many low-resource settings. ‘Technical efficiency’ is typically defined as a ratio between a weighted sum of outputs (e.g. number immunisations provided, number of antenatal visits, etc.) and a weighted sum of inputs (e.g. human and financial resources, supplies, beds, etc.); a less than ideal efficiency, as indicated by an ‘envelope’, indicates that a health facility can potentially expand their outputs without changing the quantity of inputs used [92]. Data envelopment analysis studies have been set in India [93–95], Kenya [96, 97], Sierra Leone [98], Angola [92] and Zambia [99], and helped identify inefficiencies in health services delivery, as well as opportunities to better use existing resources. Other studies exploring efficient resource allocation included a simulation model used to provide insight into better resource utilisation (e.g. personnel and physical resources) in an emergency department in Malaysia [100], and an optimisation model used to explore the optimal allocation of resources in a region in Tanzania given different health objectives (e.g. minimise number of deaths, minimise disease incidence, minimise loss of quality of life, etc.) [101].

One area of health systems and operations that was not often studied using OR was medical equipment and health technology management. In addition to two previously mentioned studies about increasing vaccine cold storage equipment [47, 91], we identified just nine OR studies related to medical equipment. Some examples include a cost-utility analysis of introducing PET scanning technology for lung cancer diagnosis in Iran [102]; a DES model of a mammography clinic in Brazil that took into account equipment failures and maintenance [103]; a queuing model developed to improve response and turn-around time of equipment repair work orders in a clinical engineering department in Cuba [104]; and models to help inform general medical equipment purchasing [105] and replacement schedules [106] in LMICs.

#### Clinical medicine

Most clinical medicine studies were focused at the national level. Common themes were assessing the cost-effectiveness of adopting new treatment or diagnostic strategies, comparing outcomes or cost-effectiveness of competing treatment options, and estimating the benefits of scaling-up treatment access.

Almost 45% (168) of studies in this category were related to HIV/AIDS, malaria or tuberculosis (TB). For example, STDSIM [107] – a microsimulation decision-support model – has been used in several studies to analyse the impact of expanding anti-retroviral (ARV) access [108, 109] as well as treating other curable sexually transmitted infections in order to prevent HIV infection [110–112]. Shillcutt et al. [113] used a decision-tree model to evaluate the relative cost-effectiveness of presumptive treatment, field standard microscopy, or rapid diagnostic tests for malaria diagnosis in different sub-Saharan African settings. A combination decision analysis and Markov model was used by Mandalakas et al. [114] to compare the cost-effectiveness of different TB prevention strategies using WHO-recommended isoniazid preventive therapy for children in close contact with infectious TB cases.

Stochastic models, such as Markov models, were common methods for clinical studies, representing almost 26% of the studies in this category. Such models are useful for simulating cohorts of patients with a specific illness as they transition from one disease state to another throughout the course of an illness or even their lifetime. For example, the cost-effectiveness of different treatment options for patients with chronic hepatitis B was studied using Markov disease models in China [115, 116], Brazil [117, 118], Turkey [119] and India [120], over time horizons ranging from 20 to 40 years.

Interestingly, 53 of the 70 studies related to the diagnosis or treatment of cancer, cardiovascular disease or diabetes were published in the past 5 years (between 2009 and 2014), consistent with increased global attention on such non-communicable diseases in LMICs [121, 122]. For example, a Markov model was developed to compare the cost-utility, in terms of quality-adjusted life years, of four different treatment options for lung cancer in Thailand [123]. DES models were used to analyse the cost-effectiveness of saxagliptin as a treatment for type II diabetes in both Argentina [124] and Brazil [125]. The treatment of mental health issues is one area that has not been studied extensively with OR – we found only 13 studies in the clinical medicine category that focused on mental illnesses in LMICs such as depression and schizophrenia.

#### Public health

Public health, specifically at the national level, was the most common global health area explored using OR. Vaccination policies, particularly the introduction of vaccines into routine child immunisation programmes, and other disease prevention strategies such as screening programs (e.g. for cervical cancer), were among the most common types of problems explored.

An example vaccination model is the TRIVAC decision-analysis model from the Pan American Health Organization ProVac initiative, which was used to assess the cost-effectiveness of adding vaccines (e.g. pneumococcal conjugate vaccine, Hib and rotavirus) to the routine child immunisation schedule in LMICs, particularly in Latin America [126, 127]. Among preventative public health measures, studies exploring screening and/or vaccination combinations were common. For example, Demarteau et al. explored efficient combinations of cervical cancer prevention strategies (e.g. screening and/or vaccination against human papillomavirus) using a combination Markov and optimisation model, in both Brazil [128] and Nigeria [129]. The Markov model estimated the costs and outcomes of different strategies, which was used as input to an optimisation model that determined the combination of prevention strategies that minimised cervical cancer cases for a fixed budget.

Similar to the clinical medicine category, HIV/AIDS, malaria and TB were a common focus for public health studies, with approximately 30% of all studies in this category dedicated to these illnesses. Simulation platforms, such as OpenMalaria [130] and STDSIM [107], have provided the modelling foundation for several public health-oriented OR studies related to such illnesses, at both a national and regional level. STDSIM was used to analyse focused public health interventions for high risk groups such as commercial sex workers [131, 132]. The OpenMalaria model was used to simulate the impact of interventions such as indoor residual spraying in the highlands of western Kenya [133].

Global-level studies represented only 2% of studies, and most of these (52%) were in the public health category. Examples of such studies include a model to recommend the required size and resulting cost of an international stockpile of cholera vaccine to enhance efforts to mitigate cholera outbreaks in the wake of natural disasters [134], and a comparison of the potential impact of rotavirus versus human papillomavirus vaccination across 72 countries eligible for support from the Global Alliance for Vaccines and Immunization (GAVI), taking into account affordability, cost-effectiveness and distributional equity [135].

One area of disease prevention that lay at the intersection of clinical medicine and public health is the prevention of mother-to-child transmission of HIV. Although some prevention strategies are of a clinical nature (e.g. administering ARVs or nevirapine), we considered this a public health issue as there are other behavioural considerations as well (e.g. recommendations for early weaning or avoidance of breast-feeding). Examples of such studies include a DES model used to evaluate relative benefits of ARVs at childbirth and/or bottle-feeding in Tanzania [45], a mathematical model comparing different feeding recommendations (i.e. exclusive replacement-feeding versus exclusive breast-feeding for durations of 4 or 6 months) at different compliance levels in Uganda and Kenya [136], and simulation studies exploring the cost-effectiveness of implementing the WHO’s 2010 guidelines for the elimination of mother-to-child transmission in Zimbabwe [137, 138].

#### Health innovation

Innovation was the least studied category of global health-related OR, with only 47 papers. The majority of these studies (89%) were related to vaccines, either in the early phases of clinical trials or yet to be developed, and were predominantly focused on HIV and malaria. Common themes were modelling the potential impact of imperfect or partially effective vaccines [139–143] or vaccines with rapidly waning protection [144, 145], modelling changes in behaviour (i.e. adopting riskier or relaxed behaviour) with the introduction of a newly developed vaccine [146–149], modelling the cost-effectiveness or willingness-to-pay thresholds of a new vaccine [150–157], forecasting demand for a new vaccine [158], or combinations of these issues [159–165].

Some studies explored the best ways to implement or roll-out a new vaccine should it become available (e.g. through the Expanded Programme on Immunization (EPI), school-based programmes, mass vaccination campaigns, targeted high risk groups, planning for follow-up boosters, etc.) particularly in cases where initial supplies are expected to be limited [166–171], as well as how a partially effective vaccine would measure up against existing prevention strategies [172] (e.g. male circumcision in the case of HIV [173] or insecticide-treated nets in the case of malaria [174]). Lee et al. [175] used a DES model of Niger’s vaccine supply chain to analyse the impact of developing thermostable versions of six currently available EPI vaccines, an innovation that could relieve bottlenecks in the cold chain. They found that thermostable versions of any of the EPI vaccines, either individually or in combination with other vaccines, would decrease cold storage and transport utilisation and increase the availability of all vaccines, even non-thermostable ones. Levin et al. [176] also explored thermostable vaccine introduction in Cambodia, Ghana and Bangladesh – their model was a spreadsheet-based decision tree and costing analysis.

Other studies examined innovations in drugs and new diagnostic technologies [177–179]. For example, Dowdy et al. [178] used a decision analysis model to estimate the cost-effectiveness of a novel point-of-care TB diagnostic tool in comparison to existing methods in South Africa, Brazil and Kenya. Cost-effectiveness was sensitive to the specificity and cost of the new test, but its introduction was estimated to avert almost 50% more disability-adjusted life years per 1000 TB suspects [178].

The examples provided in this section highlight how OR can be a useful tool for informing health policies and decision-making in low-resource settings – from studies with local health facility-level implications to analyses that are global in scope, exploring issues that span all application areas of global health. We have highlighted areas where there has been a strong OR focus; for example, national-level studies focused on clinical and public health and studies about infectious diseases such as HIV/AIDS, malaria and TB. Areas where OR analyses have been lacking include health technologies and non-vaccine-related innovation, and non-communicable diseases such as cancer, diabetes and mental health.

### Health equity theme

This section focuses on an important goal of global health – achieving equity in health for all people worldwide. The challenge in studying health equity is that there is no single way to identify or measure it within a community or population. We felt it would be compelling to discuss how issues of health equity have been analysed using an OR approach, especially given this challenge.

Out of the 1099 papers included in this review, we identified 44 studies that considered health equity as an important part of the research question being explored. Due to our review’s focus on healthcare provision and public health, rather than wider social determinants of health, the studies in this section are primarily focused on healthcare equity, specifically as it relates to socially disadvantaged groups. These studies spanned all four application areas of global health (health systems and operations (*n* = 16), clinical medicine (*n* = 4), public health (*n* = 22) and innovation (*n* = 2)) and all target levels (local (*n* = 6), national (*n* = 29), regional (*n* = 3), global (n = 4) and general (*n* = 2)). Geographically, studies were predominantly focused on South Africa (*n* = 10), China (*n* = 5) and India (*n* = 4); all other locations had just one or two studies.

Studies differed in how they operationalised (i.e. defined the measurement of) healthcare equity. Some studies defined inequity as a quantifiable disparity in a specific health indicator across different social groups (e.g. mortality risk across wealth quintiles [180], malaria incidence in children and pregnant women vs. adults [181]) and estimated how this indicator might change with a more equity-centred approach to an intervention. Other studies parameterised equity as a model variable that ranged between two extremes – from least to most equitable (e.g. percent coverage of an intervention [182, 183], measures of spatial accessibility [184] or a modified Gini coefficient [185]) – allowing researchers to explore the circumstances under which this parameter was less than ideal or even how to maximise it. Some applied a single ethical principle when operationalising equity (e.g. Wilson et al*.* [183] took an egalitarian approach), whereas others explored their research question through multiple ethical lenses [185, 186].

**Fig. 7:**
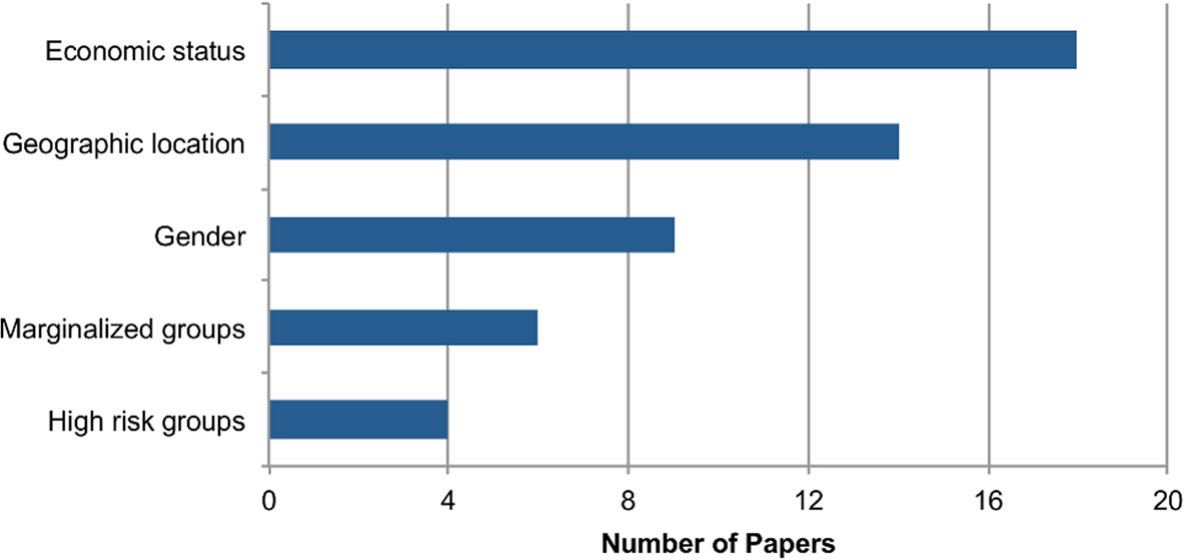
Number of equity-themed OR papers by topic area. Note that some studies looked at equity across several categories; these were counted for each relevant category. Marginalised groups include people living with HIV or other stigmatised illnesses. High risk groups include men who have sex with men, commercial sex workers, or people considered to be in high risk age groups for certain diseases

We also looked at the distribution of healthcare equity studies across groups with different levels of underlying social advantage/disadvantage, including wealth, geographic location, sex or other social status (Fig. 7).

Accessibility of healthcare, for both the financially and geographically disadvantaged, was a common theme among equity-related papers. The impact of health insurance and/or universal coverage [185, 187–189], user fees [190] and subsidies [191] on equitable healthcare accessibility and affordability was one of the most prominent themes. For example, Waters et al. [185] used a statistical probit model to analyse the potential impact of a health insurance program and various insurance eligibility standards on both overall access to healthcare, as well as equitable access to healthcare across all economic quintiles in Ecuador. Economic status was also considered by Pagel et al. [192], who explored how different community-based strategies to prevent post-partum haemorrhage affected women of different economic quintiles in Malawi, and Carrera et al. [193], who showed that an equity-focused approach to child health that prioritises the poorest and most marginalised populations could lead to higher decreases in child mortality while being more cost-effective than traditional approaches.

Geographic accessibility and distribution of health services, and the identification of geographic disparities in health, were explored by a number of resource location-allocation studies [183, 184, 186, 194–196]. For instance, Moore and Stamm [184] built a location optimisation model for cholera treatment facilities in Haiti, using the Enhanced Two-Step Floating Catchment Area method. They present their model with five unique objective functions, including one that minimises inequitable access, in order to explore the trade-offs between adequate, equitable and efficient coverage of treatment centres. Similarly, a resource allocation model of a Zambian health service delivery program parameterised equity in the objective of their optimisation model for decision-making based on resource efficiency and equity across varying geographic locations [186]. In India, a location-allocation model was used to propose new health facility locations for improved geographic access to healthcare [194].

Health equity issues related to sex [146, 197–204], high-risk groups (e.g. commercial sex workers) [205–207] or marginalised groups (e.g. people living with HIV) [182, 208–212] were commonly associated with health issues such as HIV and cardiovascular diseases. For instance, upon recognising that women often lack the power to negotiate safe sex in developing countries and can be exposed to HIV against their will, studies have analysed the effects of post-circumcision changes in male condom use [198, 200] and women-initiated vaginal microbicides [199] on gender health equity in Southern Africa. The issue of high HIV burden among sex workers was analysed using a deterministic model that compared the impact of several interventions, including equitable access to ARVs and community empowerment programs that educate female sex workers about preventive measures against HIV [207]. Two studies applied an equity lens to mathematical optimisation problems exploring optimal HIV treatment strategies in South Africa [182, 183]. Wilson et al. [183] formulated an ‘equity objective function’ to propose ARV allocation strategies that would ensure each individual infected with HIV has an equal chance of receiving ARVs. Cleary et al. [182] parameterised the concept of health equity as the percent coverage of treatment in HIV/AIDS patients. By placing different constraints on this parameter in the model, they were able to highlight the trade-off between maximising equity versus maximising health outcomes, where the ‘opportunity cost’ is QALY’s forgone in the former scenario, and higher proportions of unmet need in the latter [182].

These examples highlight the utility of OR for informing equitable health policy decision-making in low-resource settings. Equity is not a concept easily measured, nor will it be possible to achieve consensus on how it should be measured. A major contribution of OR is that it allows for equity to be quantified in different ways, often within the same modelling framework, such that trade-offs and consequences can be explored more systematically, opening up important discussions about how best to reduce systematic disparities in health for all people worldwide.

### Impact theme

In this section, we highlight seven OR studies in which the authors described how their work was implemented or was influential to specific health policy changes or decisions. This compilation is not an exhaustive list; however, studies describing implementation or impact represent a very small fraction of all papers in this review (we estimate less than 10% based on our review of abstracts). In the sub-section that follows, we explore several features of these studies that may have helped contribute to the effective translation of model recommendations into policy or practice, and discuss barriers and challenges to bridging the gap between operations research and health policy.

#### Case examples of OR impact

The first four studies are examples of impact at the national or global level. Dowdy et al. [213] used a decision tree model to estimate the cost-effectiveness of serological testing for active TB in India. Serological tests are widely used in India and other developing countries because they are fast, simple and readily available; however, no international guidelines recommend their use over other diagnostic tests such as sputum smear microscopy. The study found that serology tests can result in more secondary infections and false-positive diagnoses, and cost more per-patient, compared to sputum smear microscopy. Their findings, which were presented to a WHO Expert Group on TB in 2010, were influential to the WHO’s subsequent policy statement recommending against the use of commercial serological testing for active TB [213].

Hutton et al. [38] developed a combination decision tree and Markov model of hepatitis B infection and progression, which compared options for hepatitis B screening, vaccination and treatment in the United States and China. In China, they found that providing catch-up vaccination for children under 19 would improve health outcomes as well as save healthcare costs in the long run due to the number of infections averted. Their modelling work in 2008 was influential in China’s decision to expand free catch-up vaccination to all children under 15 in April 2009 [38].

In the wake of a global debate to shift the significant resources being used for polio eradication towards effective control [214], a systems dynamics disease outbreak model for polio developed by Thompson and Tebbens [215, 216] demonstrated that shifting to a control strategy would not only be more costly in the long run, but would lead to more cumulative cases as populations become more susceptible to new outbreaks [215]. After the results of their model were presented to global stakeholders at a WHO-convened consultation in 2007, experts were convinced that efforts towards completing eradication must continue; for example, the director of the global polio-eradication initiative at the WHO in Geneva commented that Thompson’s work put “*a nail in the coffin for the idea that there is a cheap and painless way out*”, and a representative from the global immunisation program at the United States Centers for Disease Control and Prevention commented that this analysis showed there is no viable control option and that we need to intensify eradication efforts [217]. As eradication efforts continue today, there is hope that complete eradication can be achieved in 2016; in 2015, there were fewer cases in fewer countries than ever before, and in January 2016, India marked its fifth year without a case of polio [218].

A DES model was developed by Langley et al. [39] to evaluate the impact of automated nucleic amplification test (aNAAT), a new TB diagnostic test, compared to existing techniques in Tanzania. The model recommended several combinations of TB diagnostic options incorporating aNAAT testing that were cost-effective in both urban and rural settings. At the time of publication, policymakers in Tanzania were considering specific sites for a trial of the new aNAAT technology, and results from the DES model were going to be used to inform the implementation plan for the trial [39].

The following three studies are examples of implementation on a more local level. Cruz et al. [104] developed a queuing simulation model to help enhance medical equipment repair service quality for the clinical engineering department of a 600-bed hospital in Cuba. Simulation results showed that service quality enhancements (i.e. reduced work order backlogs and service times) could be achieved without hiring new personnel. Clinical engineering management implemented two proposed strategies and major service improvements were observed over a 2-year period, as predicted by the model [104]. Perez et al. [59] used a combination DES-optimisation model to reduce wait times in the admissions centre of a health centre in Colombia with relatively low additional cost. The solutions proposed by the model were subsequently implemented, and although not explicitly measured, experts in the admissions centre noticed relevant improvements in wait times [59]. Finally, Friedrich et al. [72] developed a decision support system (DSS) using linear programming to upgrade the nurse scheduling process at a hospital in South Africa in order to improve the quality of healthcare and nursing services. The model’s objective was to minimise nurse dissatisfaction by better taking into account nurse preferences. Although the system had not been fully implemented at the time of publication, feedback provided through user validation was positive and enthusiastic. Staff managers reported that, in just a few seconds, the system performs the same time consuming computations they carry out manually each month, with improved nurse utilisation and reduced overtime [72].

#### Factors contributing to success in ‘bridging the gap’ between OR and impact

Based on these cases, three key drivers for bridging the gap between OR and impact have emerged, namely (1) engagement of local or expert stakeholders in model design and validation, particularly those in policy- or decision-making roles; (2) use of contextually representative data; and (3) a concentrated effort on communication of research findings. All selected cases demonstrated all three of these key drivers even if not explicitly cited in the discussion that follows.

##### (1) Local or expert stakeholders involved in OR model design and validation

Active participation of local stakeholders has been suggested by others as a key to strengthening health research and policy linkages [219, 220] and the examples provided in this section are evidence of this in the field of OR. Such collaborations are important for several reasons. First, the engagement of stakeholders facilitates the identification of relevant and appropriate global health research questions. Thompson and Tebbens advise that “*modelers need to focus on working effectively with the people who need and can use the results*” [215]. Langley et al. [39], who underwent a comprehensive review of the questions that policymakers need to address when assessing different TB diagnostic strategies, are a great example of this. Situating their work within this ‘Impact Assessment Framework’ [221] not only helped identify the questions that their model should answer, but also informed the appropriate choice of modelling methodology to achieve their goals. Identifying relevant research questions is perhaps more easily accomplished for OR studies based on local settings. For example, Friedrich et al. [72] conducted a root cause analysis of challenges faced at the hospital they wanted to help, and developed their decision-support solution in response to several identified problems around nurse scheduling. Cruz et al. [104] and Perez et al. [59] also worked with local collaborators to identify and model relevant problems for the health facilities they worked with.

Second, it is critical to involve local or expert collaborators in the model conception and design because they are intimately knowledgeable about the context, and can help ensure that the model and analysis accurately describes the health issue and addresses the policy questions or decisions they face. This type of collaboration was demonstrated by Hutton et al. [38], who formed a multi-disciplinary team, including the director of Stanford’s Asian Liver Centre, for their research on hepatitis B in China. They also used an iterative approach to model development, beginning with a very simple representation of patient health states, with details added incrementally based on suggestions from experts until they were satisfied that their model appropriately represented the policy problem [38]. Friedrich et al. [72] also underwent an iterative design process, whereby their nurse scheduling DSS model was tested with users and continuously improved throughout development. Langley et al. [39] worked with experts from Tanzania and Malawi to ensure the input parameters and control logic for their DES model were valid.

Third, engaging local stakeholders throughout the development process can also facilitate trust-building and implicitly lead to capacity building, helping to address the lack of technical ability to interpret findings common in LMICs [219] and empower the policymakers to take ownership of the process. For example, as a result of the work by Langley et al. [39], policy advisors in Tanzania have requested the ability to be able to use the simulation model themselves to evaluate alternative diagnostic strategies in the future. A pilot study is underway to demonstrate whether the model is sufficiently user friendly for this type of use [39]. In the case of Friedrich et al. [72], the users were pleased that their input was used so extensively in the development of key features of the nurse scheduling DSS, including the interface design and the data validation function that prevents them from entering invalid data; users even requested further training in using the DSS [72].

If collaboration between local policymakers, researchers and implementers is important for impact, then the lack thereof can be a major barrier to impact. Yet, much of the OR literature reviewed did not have a collaborator or partner in the context where their model was intended to serve. Although some studies did express a desire to use their models and results as a basis for further research in partnership with healthcare organisations, in general, few of the studies in this review mentioned showing (or even the intention of showing) their model or results to relevant stakeholders.

##### (2) Use of contextually representative data

In addition to having relevant stakeholders involved in the model conception and design, contextually representative data is also likely an important factor in generating OR analyses that have impact or are implementable. The featured case studies are examples of the use of appropriate data. The studies by both Thompson and Tebbens [216] and Dowdy et al. [213] were focused on India, and to the extent possible, were populated with input data relevant to the Indian context, from state-level statistics on population, polio incidence, etc. [216] to costing data obtained from local labs [213]. These nationally-focused studies were compelling enough to capture the attention of global stakeholders, such as the WHO, leading to broader global implications. Hutton et al. [38] conducted a comprehensive review of over 250 published papers to populate the data for their model on hepatitis B in China. Input data for the TB diagnostics model in Tanzania came from a range of sources, including the National TB and Leprosy Programme, diagnostic centre laboratory records, and local managers [39].

For locally-focused studies, especially when local collaborators are involved in the research exercise, it is often possible to prospectively collect the data required for modelling. For example, Cruz et al. [104] used data collected in the hospital’s electronic technology management system over a 3-year period for both the development of their equipment service simulation model and for the validation of their model recommendations post managerial improvements. For the study to reduce wait times at a health centre in Colombia, the time between patient arrivals and service time in the admissions centre were collected for a short period of time in order to build the simulation model. The model was further validated using admissions data provided by health centre managers [59].

For many of the other studies in this review, approximations and assumptions were required to estimate certain model parameters. Often researchers had to resort to the use of unrepresentative data, for example data from neighbouring regions or developed countries. These data assumptions and compromises, which are often unavoidable, should be taken into consideration when applying model results and recommendations to a given context.

Availability of reliable data is one of the challenges that sets low-income countries apart from middle-income countries with regards to modelling [32]. In this review, the studies in some middle-income countries (e.g. China [38] and Brazil [43]) were able to make use of centralised hospitalisation information systems and national household surveys, enhancing the validity and robustness of their models and analyses. In fact, access to data may be a reason for location selection on the part of researchers, possibly explaining our finding that low-income countries are less often targets for OR. For example, one study stated “*India was selected for this simulation because it is one of the largest developing countries and sufficient data on breast cancer epidemiology to construct a reliable and valid model were available*” [222].

##### (3) Emphasis on communication of research findings

Publication is important, but not sufficient, for the effective communication of research findings, whether from OR or other types of analyses. Communication in various forms beyond the journal publication was an important part of the case studies that influenced change. Perhaps the best example of this was Thompson and Tebben’s work on polio eradication [215], which they had the opportunity to present at a WHO stakeholder meeting in early 2007. For their presentation, they did not focus on explaining the model, equations, and diagrams in detail, but communicated the key insights in the simplest way possible [215]. For the study on TB serology testing, two of the authors were affiliated with the Stop TB Partnership’s New Diagnostics Working Group and had the opportunity to present their study findings to a WHO expert panel on TB serological testing, an audience that would be receptive to their work [213]. One study was actually translated into another language [38], and for another [72], user validation interviews were conducted in the local language in order to get the most accurate feedback possible from users.

Many of the OR models had user-friendly interfaces or used visual simulation environments as a means of communicating their model applications and results in a more personalised or accessible fashion. For example, Hutton et al. [38] specifically used Microsoft Excel because their intent was to develop a model that could easily be shared with policymakers. They “*incorporated sufficient detail to capture important characteristics of hepatitis B disease progression and treatment so that the model would be believable to a clinical audience*” [38] but tried to keep it simple enough so that those who lack modelling expertise could easily understand it. Langley et al. [39] also stressed the importance of a visual representation of the modelled processes in order to improve engagement and assist in the validation of their model with experts in Tanzania; the simulation software they chose afforded this possibility. The output of the nurse scheduling DSS model was formatted similarly to the hospital’s previous manual scheduling process so that unit managers would more easily adopt and transition to the new system [72].

Overall, the continued expansion of research reach and influence requires sustained efforts to communicate findings through different channels, with engaged outreach to, and personal connections with, policymakers and public health officials.

## Discussion

“*Scoping studies aim to map the literature on a particular topic or research area and provide an opportunity to identify key concepts; gaps in the research; and types and sources of evidence to inform practice, policy-making, and research*” [35]. The goal of this scoping review was to provide a broad overview of the use of OR in global health, with several concrete examples showing the breadth and depth of how this field of research is being applied to important global health challenges worldwide. We also explored the theme of health equity, demonstrating the unique opportunities the field of OR can contribute to this increasingly important area of global health. Cases where OR has had an impact on policy- or decision-making were also highlighted, with examples ranging from the implementation of local-level changes related to the day-to-day operations of health facilities, to decisions about national vaccination policies, to influencing international WHO policies and global perceptions about disease eradication. These cases serve as excellent examples of the importance of collaboration, data and communication for affecting change at the local and global level.

### Limitations and challenges of the review

We faced some challenges and limitations when conducting this review. Given the broad interpretations of what constitutes OR, a lack of consistent terminology for OR, and the variety of journals where OR literature in healthcare tends to be published, our search terms were broad in reach and scope, with the consequence that a large amount of literature was captured that was not relevant. Additionally, the geographic search tailored to LMICs was not straightforward; country names are not always considered controlled vocabulary, so every individual country name had to be included as keywords.

We also had to be pragmatic at the outset about the coverage of the review. We chose to focus on papers published in the year 2000 and later. As such, there is some overlap with the hand-searched literature [11, 16, 17, 23, 24, 26–28, 32], but not with the review of OR in global health by Datta [10], which at the time of our review was over 20 years old. To fill this gap would have been an onerous task. Due to rapid advancements in computing technology, it could be argued that OR models developed before 2000 are out-dated, as are any global health data used to populate them. Further, we also had to be selective when setting the inclusion criteria for the types of ‘OR’ and ‘health’ studies explored given these terms have such broad definitions. We restricted OR studies to those where modelling or analytical methods were used with an orientation towards decision-making. Our health focus was largely on healthcare provision and public health, and did not include the wider social determinants of health; this focus was inherently reflected in the subset of studies explored for the health equity-theme as well. As it was, due to the volume of literature included, we could not summarise or cite all of the studies found; however, we hope this review has provided enough of a landscape overview to prompt further exploration of the utility of OR in this context. A complete database of the 1099 studies is provided as Additional file 2.

Income categories for countries were based on 2014 World Bank classifications, regardless of how a country may have been classified historically. It would have been difficult to track shifts in income classification for every country and every paper included in this review. Furthermore, we felt the interpretation would be simpler knowing that all studies related to a specific country (e.g. Brazil) were consistently counted towards its current income category (e.g. upper-middle) rather than split across multiple categories.

Our criteria for ‘impact’ when selecting case examples was that the study meaningfully informed a policy decision or the recommendations were implemented in a real-world setting. We were unable to make any inferences about the magnitude of improvement in health that may result from these changes. Only Hutton et al. [38] presented estimates that 170 million children would be vaccinated for hepatitis B in China as a result of their model recommendations, preventing almost 8 million infections and 70,000 deaths, and saving the equivalent of $1.4 billion over the lifetime of these children.

A final limitation of this review was the restricted search for published literature alone. Those papers that did not describe a particular policy change could have indeed influenced decision-making after their publication. Future work could involve searching grey literature for case studies and policy documents suggesting that knowledge gained from OR was influential in decision-making processes. The lack of published evidence that OR is impacting policy change represents a major missed opportunity for the academic community to learn and better engage in impactful OR work. It has been mentioned that fora are needed where these findings can be discussed [219] and success stories of policy transfer shared with a broader community [14], if not in peer-reviewed literature, then elsewhere.

In general, scoping reviews take a considerable amount of time and skill. Balancing feasibility, breadth and comprehensiveness can be a challenge given available time, funding and resources [35, 37]. Although not explicitly tracked, we thought it would be informative to provide an estimate of the amount of time that it took to conduct our review. The most time intensive stages were stages 2 to 4 – designing the search, identifying and selecting studies and charting data. Stage 2 was conducted over a period of approximately 4 months by one author on a near full-time basis (estimated 500 person-hours), and stages 3 and 4 were carried out by four co-authors over a period of approximately 8 months, all on a part-time basis (estimated 340 person-hours). This is consistent with the findings of Pham et al. [36], who reported scoping reviews have taken anywhere from 2 weeks to 20 months to complete.

### Global overview and global health application areas

Despite these limitations and challenges, this scoping review, consisting of 1099 studies, is to our knowledge the most comprehensive review of OR in global health to date. Our overview highlighted that low-income countries are less frequently studied using OR compared to middle-income countries, a trend that does not seem to be improving with time. Furthermore, a large proportion of healthcare-related OR in LMICs has focused on just six middle-income countries. If population is an appropriate yardstick for research focus, then perhaps this representation is reasonable; however, the disparity between volume of literature and number of countries in each income category is much more pronounced. That being said, 84 LMICs around the globe have been the focus of at least one OR study since 2000; hopefully, increased global coverage will continue.

Although the aggregate data did not show that any particular OR method was dominant, we found that certain types of research questions were more amenable to specific OR methods than others (e.g. local and national-level health systems problems were commonly studied with simulation or optimisation methods). This highlights the importance of a collaborative and interdisciplinary approach to applying OR in global health, such that those with modelling expertise in specific methods can apply their expertise where it can make the most impact.

We found that the majority of OR literature explores clinical medicine and public health issues at a national level, and that, although very helpful for exploring the impact of interventions on a macro scale or adding to discussions on global priority-setting, fewer studies were targeted at the regional or global level. Since OR models tend to describe the dynamics and interdependencies of actual systems, it likely gets more difficult to develop accurate models as complexity increases (i.e. from local or national systems to regional or global systems). OR models are also highly dependent on input data, which is likely easier to obtain at the local and/or national level. Studies targeted at several countries or a whole region have themselves cautioned that more specific country-level analyses with more representative data are needed for country-level decision-making, which is perhaps why few studies are targeted at these levels [135, 193].

One area of global health that OR has made a unique contribution to is the analysis of new health innovations. This small subset of studies highlights the value of OR for analysing global health interventions that cannot yet be trialled or implemented on the ground because the scientific breakthroughs have yet to be achieved. Understanding the potential impact or possible implementation challenges of new innovations is important to their successful roll-out when they do become available. OR can also help highlight important design criteria targets for the development of new innovations, in terms of both cost and minimum levels of efficacy or specificity required to achieve desired outcomes.

Infectious diseases (e.g. HIV/AIDS, malaria, TB) continue to be a major focus of OR globally; however, there has been an increase in the number of OR studies about non-communicable diseases over the past 5 years. Neglected areas representing an opportunity for future OR include analyses focused on low- and lower-middle income countries, non-communicable diseases (particularly mental health), and medical equipment and technology planning.

Additional study characteristics that could have been analysed include the funding sources, the academic institutions of the lead investigators and the quality of the studies themselves. Funding sources have been identified as a potential external influence on both research and policy agendas, but in some cases resources dedicated to an OR study can lead to positive change. For example, two studies from this review [43, 223] were funded by the Brazilian Ministry of Health [223], indicating interest on the part of the government to use OR as a tool to answer a key health systems-related questions. Exploring funding sources in more detail could be an area of future consideration. The quality of the studies themselves could also be highly relevant to successful research uptake, but we did not undertake critical appraisal for this review as quality assessment does not typically form part of the scoping study remit [34]. Others have reported on the quality of simulation models in healthcare by applying strict quality criteria during the review process [18].

### Health equity: an opportunity for future OR

The healthcare equity-themed studies featured in this review demonstrate the utility of OR for informing equitable health policy decision-making in low-resource settings. These studies, however, represented a relatively minor proportion (4%) of all global health-related OR in the time period studied. Some have argued that a major shortcoming of the Millennium Development Goals was a failure to address equity [6], and that “*looking forward, equity analyses and actions need to be an integral part of programme strategies rather than an afterthought*” [7]. We believe a huge opportunity exists to apply the tools and techniques of OR to study health equity in the post-2015 era.

First, OR is extremely versatile in how the concept of health equity can be operationalised. The examples in this review demonstrate a variety of different ways to quantify equity - from measured disparities in specific health indicators to parameterised model variables. Although beyond the scope of this review, these principles can be used to explore the broader social determinants of health as well. As no single measure is sufficient to assess inequities, those applying OR to health equity could benefit from integrating established health equity frameworks into their approach (e.g. the PROGRESS [224, 225] and PROGRESS-Plus [226] frameworks) to help ensure the explicit consideration of important equity factors in the design of OR models and analyses. Second, OR allows for the comparison of different equity goals (e.g. using different ethical principles or comparing efficiency versus equity), often within the same modelling framework. For example, a utilitarian perspective aims to maximise overall societal benefit, whereas an egalitarian approach would strive to achieve equal distribution of, or access to, resources for every person [183]. OR allows for a more systematic analysis of the trade-offs and consequences of viewing equity from these different perspectives. Third, OR models can study the effect that different policies or decisions might have on marginalised populations without the ethical implications of a real-world study. Arguments could be made that, to achieve health equity, certain groups should be valued over others; for example, perhaps high-risk groups or the least advantaged should be prioritised, rather than treat all cases alike regardless of social standing. OR can aid in testing such sensitive policy hypotheses a priori without unintentional consequences.

OR can be applied at national and sub-national levels, across different socioeconomic groups and marginalised populations, and through different ethical lenses, in order to inform interventions and health policy decisions that will promote better equity in health going forward. We hope the equity-themed literature highlighted in this review can help open up important discussions about how best to model and analyse systemic disparities in health for all people worldwide.

### OR impact: recommendations for bridging the gap between OR and policy or practice

Studies describing OR implementation or impact represent a small proportion of the literature reviewed – there is still a far way to go for OR to reach its full potential in global health. Our finding that few papers present details of implementation or impact is consistent with the experience of others who have reviewed OR in developing countries [10, 11, 23, 24]; for example, Datta [10] reported that less than 5% of studies reviewed discussed implementation. From the numerous studies included in this review that did not appear to have any influence on policy or decision-making, several persistent challenges emerged as common themes, including lack of local or expert stakeholder engagement in model conception and design, challenges in acquiring reliable and representative data, and a lack of communication strategy beyond the journal publication.

The lack of appropriate packaging of research findings or exclusive dissemination within academic circles was also found by others to be barriers to research uptake in LMICs [219, 227]. In recent years, several theories, frameworks and practical handbooks or ‘toolkits’ have been developed by agencies such as the Overseas Development Institute [228–230], the International Development Research Council [231], the WHO [232], and the Institute of Development Studies [233], to help guide and make more effective the translation of research into policy. Specifically, there is a focus in this literature on the effective communication of research findings, which extends beyond just communication products (e.g. policy brief, stories of change, etc.) to a whole body of research on knowledge sharing, knowledge transfer and knowledge translation. The use of packaging and language that are more appropriate and targeted towards implementation can help enhance the impact of OR.

Yet, there have been many success stories of global health research effectively bridging the research-policy gap [13, 234]. For example, Zachariah et al. [13] identified several impactful operational research studies that had implications for policy and practice; however, all of these studies were field studies that did not involve modelling and thus did not meet the inclusion criteria for this review. Additional guidance to be gleaned from these studies for the OR modelling community include (1) the generation of research questions from within existing programs, which are focused and of simple design; (2) working with partners to ensure that sufficient resources (human and financial) are available for an engaged and motivated research process that extends beyond models and analyses; (3) setting realistic expectations of research impact; (4) investing in long-term research and policymaker relationships; and (5) helping build capacity of end-users to use research to demand policy change [13, 234].

Promisingly, within the OR community, there is a growing movement towards impact-driven research and publication. The “Doing Good with Good OR” paper series and research award, offered by the Institute for Operations Research and the Management Sciences, and the “OR in Development” prize, offered by the International Federation of Operational Research Societies, are efforts to recognise OR for the impact of the analysis, in addition to its analytical rigor. In general, there is a need for incentivising the engagement of researchers in problems that are relevant and timely to important policy issues [219]. Hopefully, these efforts, paired with efforts within developing countries to increase end-user capacity to use OR [11, 25] will help bridge the gap between OR and impact in LMICs.

## Conclusion

There is a tremendous opportunity for OR researchers and global health practitioners alike to continue to apply OR in global health, particularly in areas where such studies may currently be lacking. We hope the findings of this scoping review, which represents the most comprehensive compilation of OR literature in global health to date, are of interest to a wide-ranging group of stakeholders engaged in global health policy and practice. For government bodies and administrators of health programs and services, we hope to have showcased the utility of the OR approach in modelling policy and programme changes to improve efficiency, particularly when resources are limited. We also hope funders of international development research see value in allocating funding to operations research within broader global health programs. We hope those currently engaged in OR can benefit from the impactful studies highlighted in this review, and we encourage them to share the impact of their work more broadly so that others can learn from challenges and successes.

## Additional files

Additional file 1: Search strategies for Scopus, Compendex, Inspec and HealthStar databases. *Description*: Tables containing details of custom systematic search strategies for the Scopus, Compendex, Inspec and HealthStar databases. (DOCX 96 kb)

Additional file 2: Database of references included in review. *Description*: An Excel database of the 1099 references included in the global overview of this review paper, including columns for Authors, Title, Journal and/or Conference and/or Book Title, Year, Volume, Pages, Conference location and/or Place Published, and Abstract. (XLSX 893 kb)

## Abbreviations

aNAAT: automated nucleic amplification test
ARV: anti-retroviral
DES: discrete-event simulation
DSS: decision support system
EPI: Expanded Programme on Immunization
LMICs: low- and middle-income countries
OR: operations research
TB: tuberculosis.

## Footnotes

1 The optional ‘Consultation Exercise’ of the framework was not conducted.

2 Individualised search strategies were based on whether the database was indexed by subject headings, controlled vocabulary or keywords.

3 World Bank classification as of July 1, 2014, not the year the study was published. Note that some countries may have shifted categories since 2000.

4 Based on World Bank country and population listings [235]. There were 136 low- and middle-income countries as of 2014.

## References

[1] Koplan JP, Bond TC, Merson MH, Reddy KS, Henry Rodriguez M, Sewankambo NK (2009). Towards a common definition of global health. Lancet..

[2] Braveman P, Gruskin S (2003). Defining equity in health. J Epidemiol Commun Health..

[3] UNAIDS, UNICEF, UNFPA, WHO. Health in the Post-2015 UN Development Agenda. UN: Thematic Think Piece; 2012. http://www.un.org/millenniumgoals/pdf/Think%20Pieces/8_health.pdf.

[4] D’Ambruoso L (2013). Global health post-2015: the case for universal health equity. Glob Health Action..

[5] World Health Organization (2012). Positioning Health in the Post-2015 Development Agenda: WHO Discussion Paper.

[6] Waage J, Banerji R, Campbell O, Chirwa E, Collender G, Dieltiens V (2010). The Millennium Development Goals: A cross-sectoral analysis and principles for goal setting after 2015. Lancet..

[7] Bryce J, Victora CG, Black RE (2013). The unfinished agenda in child survival. Lancet..

[8] The Institute for Operations Research and the Management Sciences (INFORMS): What is Operations Research? 2015. https://www.informs.org/About-INFORMS/What-is-Operations-Research. Accessed 14 Aug 2015.

[9] The OR Society. About OR. 2015. http://www.scienceofbetter.co.uk/about-or. Accessed 14 Aug 2015.

[10] Datta S (1993). Applications of OR in health in developing countries: A review. Soc Sci Med..

[11] White L, Smith H, Currie C (2011). OR in developing countries: A review. Eur J Oper Res..

[12] Horstick O, Sommerfeld J, Kroeger A, Ridley R (2010). Operational research in low-income countries. Lancet Infect Dis..

[13] Zachariah R, Harries AD, Ishikawa N, Rieder HL, Bissell K, Laserson K (2009). Operational research in low-income countries: what, why, and how?. Lancet Infect Dis..

[14] Bissell K, Lee K, Freeman R (2011). Analysing policy transfer: perspectives for operational research. Int J Tuberc Lung Dis..

[15] Quaglio G, Ramsay A, Harries AD, Karapiperis T, Putoto G, Dye C (2014). Calling on Europe to support operational research in low-income and middle-income countries. Lancet Global Health..

[16] Royston G (2011). Meeting global health challenges through operational research and management science. Bull World Health Organ..

[17] Rais A, Viana A (2010). Operations research in healthcare: a survey. Int Trans Oper Res..

[18] Fone D, Hollinghurst S, Temple M, Round A, Lester N, Weightman A (2003). Systematic review of the use and value of computer simulation modelling in population health and health care delivery. J Public Health..

[19] Günal MM, Pidd M (2010). Discrete event simulation for performance modelling in health care: a review of the literature. J Simulation..

[20] Zaric GS (2013). Operations Research and Health Care Policy.

[21] Brailsford S, Vissers J (2011). OR in healthcare: a European perspective. Eur J Oper Res..

[22] Fakhimi M, Probert J (2013). Operations research within UK healthcare: a review. J Enterp Info Manag..

[23] Smith DK (2011). A bibliography of applications of operational research in sub-Saharan Africa. Int Trans Oper Res..

[24] Smith DK (2008). A bibliography of applications of operational research in West Africa. Int Trans Oper Res..

[25] Caulkins JP, Eelman E, Ratnatunga M, Schaarsmith D (2008). Operations research and public policy for Africa: harnessing the revolution in management science instruction. Int Trans Oper Res..

[26] Xiong W, Hupert N, Hollingsworth EB, O’Brien ME, Fast J, Rodriguez WR (2008). Can modeling of HIV treatment processes improve outcomes? Capitalizing on an operations research approach to the global pandemic. BMC Health Serv Res..

[27] Long EF, Brandeau ML. OR’s next top model: decision models for infectious disease control. INFORMS Tutorials in Operations Research: Decision Technologies and Applications. 2009. pp. 123–38. doi:10.1287/educ.1090.0066.

[28] Stover J (2011). HIV models to inform health policy. Curr Opin HIV AIDS..

[29] Houben RMGJ, Dowdy DW, Vassall A, Cohen T, Nicol MP, Granich RM (2014). How can mathematical models advance tuberculosis control in high HIV prevalence settings?. Int J Tuberc Lung Dis..

[30] Johnson LF, White PJ (2011). A review of mathematical models of HIV/AIDS interventions and their implications for policy. Sex Transm Infect..

[31] Yadav P, Cochran JJ (2010). Improving public health in developing countries through operations research. Wiley Encyclopedia of Operations Research and Management Science.

[32] Royston G (2012). Special issue on global health. Health Care Manag Sci..

[33] Ergun O, Keskinocak P, Swann J (2011). Introduction to the special issue on humanitarian applications: doing good with good OR. Interfaces..

[34] Arksey H, O’Malley L (2005). Scoping studies: towards a methodological framework. Int J Soc Res Methodol..

[35] Daudt HM, van Mossel C, Scott SJ (2013). Enhancing the scoping study methodology: a large, inter-professional team’s experience with Arksey and O’Malley’s framework. BMC Med Res Methodol..

[36] Pham MT, Rajić A, Greig JD, Sargeant JM, Papadopoulos A, McEwen SA (2014). A scoping review of scoping reviews: advancing the approach and enhancing the consistency. Res Synth Methods..

[37] Levac D, Colquhoun H, O’Brien KK (2010). Scoping studies: advancing the methodology. Implement Sci..

[38] Hutton DW, Brandeau ML, So SK (2011). Doing good with good OR: supporting cost-effective hepatitis B interventions. Interfaces..

[39] Langley I, Doulla B, Lin H-H, Millington K, Squire B (2012). Modelling the impacts of new diagnostic tools for tuberculosis in developing countries to enhance policy decisions. Health Care Manag Sci..

[40] Binagwaho A, Pegurri E, Muita J, Bertozzi S (2010). Male circumcision at different ages in rwanda: a cost-effectiveness study. PLoS Med..

[41] Ryan M, Griffin S, Chitah B, Walker AS, Mulenga V, Kalolo D (2008). The cost-effectiveness of cotrimoxazole prophylaxis in HIV-infected children in Zambia. AIDS..

[42] Sladkevicius E, Pollitt RJ, Mgadmi A, Guest JF (2010). Cost effectiveness of establishing a neonatal screening programme for phenylketonuria in Libya. Appl Health Econ Health Policy..

[43] Sartori AMC, de Soarez PC, Novaes HMD (2012). Cost-effectiveness of introducing the 10-valent pneumococcal conjugate vaccine into the universal immunisation of infants in Brazil. J Epidemiol Commun Health..

[44] Griffiths UK (2005). The cost-effectiveness of introducing hepatitis B vaccine into infant immunization services in Mozambique. Health Policy Plan..

[45] Rauner MS, Brailsford SC, Flessa S (2005). Use of discrete-event simulation to evaluate strategies for the prevention of mother-to-child transmission of HIV in developing countries. J Oper Res Soc..

[46] Assi T-M, Brown ST, Djibo A, Norman BA, Rajgopal J, Welling JS (2011). Impact of changing the measles vaccine vial size on Niger’s vaccine supply chain: a computational model. BMC Public Health..

[47] Haidari LA, Connor DL, Wateska AR, Brown ST, Mueller LE, Norman BA (2013). Augmenting transport versus increasing cold storage to improve vaccine supply chains. PLoS One..

[48] Harper PR, Shahani AK (2003). A decision support system for the care of HIV and AIDS patients in India. Eur J Oper Res..

[49] Deo S, Topp S, Garcia A, Soldner M, Yagci Sokat K, Chipukuma J (2012). Modeling the impact of integrating HIV and outpatient health services on patient waiting times in an urban health clinic in Zambia. PLoS One..

[50] Bishai D, Colchero A, Durack DT (2007). The cost effectiveness of antiretroviral treatment strategies in resource-limited settings. AIDS..

[51] Vieira IT, Harper PR, Shahani AK, De Senna V (2003). Mother-to-child transmission of HIV: a simulation-based approach for the evaluation of intervention strategies. J Oper Res Soc..

[52] Lauria DT, Maskery B, Poulos C, Whittington D (2009). An optimization model for reducing typhoid cases in developing countries without increasing public spending. Vaccine..

[53] Chang P-L, Hsieh P-N (2008). Bibliometric overview of operations research/management science research in Asia. Asia Pac J Oper Res..

[54] Hankins C, Hargrove J, Williams B, Abu Raddad L, Auvert B, Bollinger L (2009). Male circumcision for HIV prevention in high HIV prevalence settings: what can mathematical modelling contribute to informed decision making?. PLoS Med..

[55] Dabis F, Bazin B, Delfraissy JF (2011). Implementation and operational research in francophone Africa. JAIDS..

[56] Al-Hawari T, Al-Natour M, Ababneh B, Ahmed A (2011). Improving productivity and quality of service in internal medicine clinics through simulation. Proceedings of the 2011 IIE Annual Conference.

[57] Chen BL, Li ED, Yamawuchi K, Kato K, Naganawa S, Miao WJ (2010). Impact of adjustment measures on reducing outpatient waiting time in a community hospital: application of a computer simulation. Chin Med J..

[58] Massote AA, Caruso D, Sousa JBG. Application of Discrete Systems Simulation to Reduce Waiting Time in the Outpatient Service of a Hospital in the City of São Paulo, Brazil. In: 1st International Workshop on Innovative Simulation for Health Care, IWISH 2012, Held at the International Multidisciplinary Modeling and Simulation Multiconference, I3M 2012. Vienna; 2012. pp. 156–61.

[59] Perez K, Cardona L, Gomez S, Olarte T, Escudero P. Simulation and Optimization in a Health Center in Medellin, Colombia. In: IEEE Winter Simulation Conference (WSC 2008). IEEE. 2008;2008:1362–7. doi:10.1109/WSC.2008.4736211.

[60] Song J, Chen W, Wang L (2012). A block queueing network model for control patients flow congestion in urban healthcare system. Int J Services Oper Inform..

[61] Ahmad N, Ghani NA, Kamil AA, Tahar RM. Emergency Department Problems: A Call for Hybrid Simulation. In: Proceedings of the World Congress on Engineering (WCE 2012). London: 2012. pp. 1470–4. www.iaeng.org/publication/WCE2012/WCE2012_pp1470-1474.pdf.

[62] Ajami S, Ketabi S, Yarmohammadian MH, Bagherian H (2012). Wait time in emergency department (ED) processes. Med Arh..

[63] Eskandari H, Riyahifard M, Khosravi S, Geiger CD. Improving the Emergency Department Performance using Simulation and MCDM Methods. In (2011). IEEE Winter Simulation Conference (WSC 2011). IEEE..

[64] Gul M, Guneri AF (2012). A computer simulation model to reduce patient length of stay and to improve resource utilization rate in an emergency department service system. Int J Ind Eng Theory..

[65] Assem M, Ouda BK, Wahed MA. Improving Operating Theatre Design using Facilities Layout Planning. In (2012). Cairo International Biomedical Engineering Conference (CIBEC 2012). IEEE..

[66] Cao LS, Zhang YB, Chang C, Deng DX. Evaluation Model of the Lay-out of Sickbeds that Based on the Overall Satisfaction. In: 2010 IEEE International Conference on Advanced Computer Control (ICACC 2010). Shenyang: IEEE; 2010. p. 307–11. doi:10.1109/ICACC.2010.5486949.

[67] Adriana O, Alexandru C, Olimpia B. The Application of the EOQ Model in the Health Services Inventory Management using WinQSB Software. In (2010). IEEE International Conference on Information Management and Engineering (ICIME 2010). IEEE..

[68] Ganesh K, Ganesh S, Narendran TT, Muraleedharan VR (2011). Drug inventory management at public healthcare institutions – a case study. Int J Logist Econ Glob..

[69] Abdollahi SM, Ansari Z (2013). A data-driven goal programming model for the nurse scheduling problem. Int J Exp Des Process Optim..

[70] Abobaker RA, Ayob M, Hadwan M. Greedy Constructive Heuristic and Local Search Algorithm for Solving Nurse Rostering Problems. In: 2011 IEEE Conference on Data Mining and Optimization (DMO 2011). IEEE. 2011. pp. 194–8. doi:10.1109/DMO.2011.5976527.

[71] Ayob M, Hadwan M, Nazri MZA, Ahmad Z (2013). Enhanced harmony search algorithm for nurse rostering problems. J Appl Sci..

[72] Friedrich S, Van Dyk L. Developing a Decision Support System for Nurse Scheduling at a Public Hospital in South Africa. In: 42nd International Conference on Computers and Industrial Engineering. CIE42 Proceedings. Cape Town: CIE & SAIIE; 2012. pp. 15.1–15.9.

[73] Hadwan M, Ayob MB. An Exploration Study of Nurse Rostering Practice at Hospital Universiti Kebangsaan Malaysia. In (2009). IEEE Conference on Data Mining and Optimization (DMO 2009). IEEE..

[74] Jamom M, Ayob M, Hadwan M. A Greedy Constructive Approach for Nurse Rostering Problem. In: 2011 IEEE Conference on Data Mining and Optimization (DMO 2011). IEEE. 2011. pp. 227–31. doi:10.1109/DMO.2011.5976532.

[75] Bester MJ, Nieuwoudt I, Van Vuuren JH (2007). Finding good nurse duty schedules: a case study. J Sched..

[76] Cheng Y-J. An MIP Model for Surgery Scheduling in Combination with Surgeon Shift Scheduling. In: Proceedings of 20th International Conference on Industrial Engineering and Engineering Management. Baotou; 2013. pp. 963–71. doi:10.1007/978-3-642-40063-6_95.

[77] Ferreira RB, Coelli FC, Pereira WCA, Almeida RMVR (2008). Optimizing patient flow in a large hospital surgical centre by means of discrete-event computer simulation models. J Eval Clin Pract..

[78] Li L, Yang Y, Xueyong Y, Taibo L, Renrong G. Surgical Scheduling Based on Off-line Bin-packing. In: Proceedings of the 2012 9th International Conference on Service Systems and Service Management (ICSSSM 2012). IEEE. 2012. pp. 491–4. doi:10.1109/ICSSSM.2012.6252285.

[79] Ogulata SN, Erol R (2003). A hierarchical multiple criteria mathematical programming approach for scheduling general surgery operations in large hospitals. J Med Syst..

[80] Asim Azim M, Jianwu D, Yangping W, Fengwen Z (2014). Coverage based empirical modelling for EMS rescue system of Karachi (Pakistan). Tehnicki Vjesnik..

[81] Silva PMS, Pinto LR. Emergency Medical Systems Analysis by Simulation and Optimization. In (2010). IEEE Winter Simulation Conference (WSC 2010). IEEE..

[82] Coskun N, Erol R (2010). An optimization model for locating and sizing emergency medical service stations. J Med Syst..

[83] Chin-Tsai L, Meng-Chuan T (2010). Evaluating the optimal city in South China for new medical facilities: the application modified Porter’s Diamond Framework. J Test Eval..

[84] Le Roux F, Botha GJ. Health Post Location for Community Oriented Primary Care. In: 42nd International Conference on Computers and Industrial Engineering. CIE42 Proceedings. Cape Town: CIE & SAIIE; 2012. pp. 413–21.

[85] Shariff SSR, Moin NH, Omar M (2012). Location allocation modeling for healthcare facility planning in Malaysia. Comput Ind Eng..

[86] Cocking C, Flessa S, Reinelt G. Locating Health Facilities in Nouna District, Burkina Faso. In: 2005 Operations Research Proceedings. 2005. pp. 431–6. doi:10.1007/3-540-32539-5_68.

[87] Lee BY, Assi T-M, Rookkapan K, Wateska AR, Rajgopal J, Sornsrivichai V (2011). Maintaining vaccine delivery following the introduction of the rotavirus and pneumococcal vaccines in Thailand. PLoS One..

[88] Assi T-M, Rookkapan K, Rajgopal J, Sornsrivichai V, Brown ST, Welling JS (2012). How influenza vaccination policy may affect vaccine logistics. Vaccine..

[89] Lee BY, Assi T-M, Rookkapan K, Connor DL, Rajgopal J, Sornsrivichai V (2011). Replacing the measles ten-dose vaccine presentation with the single-dose presentation in Thailand. Vaccine..

[90] Assi T-M, Brown ST, Kone S, Norman BA, Djibo A, Connor DL (2013). Removing the regional level from the Niger vaccine supply chain. Vaccine..

[91] Haidari LA, Connor DL, Wateska AR, Brown ST, Mueller LE, Norman BA (2013). Only adding stationary storage to vaccine supply chains may create and worsen transport bottlenecks. J Public Health Man..

[92] Kirigia JM, Emrouznejad A, Cassoma B, Asbu EZ, Barry S (2008). A performance assessment method for hospitals: the case of municipal hospitals in Angola. J Med Syst..

[93] Dash U, Vaishnavi SD, Muraleedharan VR (2010). Technical efficiency and scale efficiency of district hospitals: a case study. J Health Manage..

[94] De P, Dhar A, Bhattacharya BN (2012). Efficiency of health care system in India: an inter-state analysis using DEA approach. Soc Work Public Health..

[95] Mogha SK, Yadav SP, Singh SP (2014). New slack model based efficiency assessment of public sector hospitals of Uttarakhand: State of India. Int J Syst Assur Eng Manag..

[96] Kirigia JM, Emrouznejad A, Sambo LG (2002). Measurement of technical efficiency of public hospitals in Kenya: using data envelopment analysis. J Med Syst..

[97] Kirigia JM, Emrouznejad A, Sambo LG, Munguti N, Liambila W (2004). Using data envelopment analysis to measure the technical efficiency of public health centers in Kenya. J Med Syst..

[98] Renner A, Kirigia JM, Zere EA, Barry SP, Kirigia DG, Kamara C (2005). Technical efficiency of peripheral health units in Pujehun district of Sierra Leone: a DEA application. BMC Health Serv Res..

[99] Masiye F (2007). Investigating health system performance: an application of data envelopment analysis to Zambian hospitals. BMC Health Serv Res..

[100] Ahmad N, Ghani NA, Kamil AA, Tahar RM, Teo AH (2012). Evaluating emergency department resource capacity using simulation. Mod Appl Sci..

[101] Flessa S (2003). Priorities and allocation of health care resources in developing countries: A case-study from the Mtwara region. Tanzania Eur J Oper Res..

[102] Sari AA, Ravaghi H, Mobinizadeh M, Sarvari S (2013). The cost-utility analysis of PET-scan in diagnosis and treatment of non-small cell lung carcinoma in Iran. Iran J Radiol.

[103] Coelli FC, Almeida RMVR, Pereira WCA (2010). A cost simulation for mammography examinations taking into account equipment failures and resource utilization characteristics. J Eval Clin Pract..

[104] Cruz AM, Rodriguez Denis E, Sanchez Villar C, Pozo Punales ET, Vergara PI (2003). Measured effects of user and clinical engineer training using a queuing model. Biomed Instrum Technol..

[105] Ramirez EFF, Calil SJ. Connectionist Model to Help the Evaluation of Medical Equipment Purchasing Proposals. In: IFMBE Proceedings of the World Congress on Medical Physics and Biomedical Engineering. 2006. pp. 3786–9. doi:10.1007/978-3-540-36841-0_958.

[106] Ouda BK, Mohamed ASA, Saleh NSK. A Simple Quantitative Model for Replacement of Medical Equipment Proposed to Developing Countries. In: 2010 Cairo International Biomedical Engineering Conference (CIBEC 2010). 2010. pp. 188–91. doi:10.1109/CIBEC.2010.5716050.

[107] Van der Ploeg CPB, Van Vliet C, De Vlas SJ, Ndinya-Achola JO, Fransen L, Van Oortmarssen GJ (1998). STDSIM: a microsimulation model for decision support in STD control. Interfaces..

[108] Hontelez JAC, De Vlas SJ, Baltussen R, Newell ML, Bakker R, Tanser F (2012). The impact of antiretroviral treatment on the age composition of the HIV epidemic in sub-Saharan Africa. AIDS..

[109] Hontelez JAC, Lurie MN, Bärnighausen T, Bakker R, Baltussen R, Tanser F (2013). Elimination of HIV in South Africa through expanded access to antiretroviral therapy: a model comparison study. PLoS Med..

[110] Korenromp EL, van Vliet C, Grosskurth H, Gavyole A, Van Der Ploeg CPB, Fransen L (2000). Model-based evaluation of single-round mass treatment of sexually transmitted diseases for HIV control in a rural African population. AIDS..

[111] White RG, Freeman EE, Orroth KK, Bakker R, Weiss HA, O’Farrell N, et al. Population-level effect of HSV-2 therapy on the incidence of HIV in sub-Saharan Africa. Sex Transm Infect. 2008;84 Suppl 2:ii12–8. 10.1136/sti.2008.029918 PMC2602752 18799486

[112] White RG, Orroth KK, Glynn JR, Freeman EE, Bakker R, Habbema JDF (2008). Treating curable sexually transmitted infections to prevent HIV in Africa: still an effective control strategy?. JAIDS..

[113] Shillcutt S, Morel C, Goodman C, Coleman P, Bell D, Whitty CJM (2008). Cost-effectiveness of malaria diagnostic methods in sub-Saharan Africa in an era of combination therapy. Bull World Health Organ..

[114] Mandalakas AM, Hesseling AC, Gie RP, Schaaf HS, Marais BJ, Sinanovic E (2013). Modelling the cost-effectiveness of strategies to prevent tuberculosis in child contacts in a high-burden setting. Thorax..

[115] Wu B, Li T, Chen H, Shen J (2010). Cost-effectiveness of nucleoside analog therapy for hepatitis B in China: a Markov analysis. Value Health..

[116] Wei L, Hu S, Hou J, Liu G, Ren H, Duan Z (2013). A novel estimation of the impact of treatment with entecavir on long-term mortality, morbidity, and health care costs of chronic hepatitis b in China. Value Health Reg Issues..

[117] Wiens A, Lenzi L, Venson R, Pedroso MLA, Correr CJ, Pontarolo R (2013). Economic evaluation of treatments for chronic hepatitis B. Braz J Infect Dis..

[118] Almeida AM, da Silva AL, Cherchiglia ML, Andrade EI, de Oliveira GL, Acurcio FA (2011). Chronic hepatitis B treatment: the cost-effectiveness of interferon compared to lamivudine. Value Health.

[119] Toy M, Onder FO, Idilman R, Kabacam G, Richardus JH, Bozdayi M (2012). The cost-effectiveness of treating chronic hepatitis B patients in a median endemic and middle income country. Eur J Health Econ..

[120] Aggarwal R, Ghoshal UC, Naik SR (2002). Treatment of chronic hepatitis B with interferon-alpha: cost-effectiveness in developing countries. Natl Med J India..

[121] Di Cesare M, Khang Y-H, Asaria P, Blakely T, Cowan MJ, Farzadfar F (2013). Inequalities in non-communicable diseases and effective responses. Lancet..

[122] Alleyne G, Binagwaho A, Haines A, Jahan S, Nugent R, Rojhani A (2013). Embedding non-communicable diseases in the post-2015 development agenda. Lancet..

[123] Thongprasert S, Tinmanee S, Permsuwan U (2011). Cost-utility and budget impact analyses of gefitinib in second-line treatment for advanced non-small cell lung cancer from Thai payer perspective. Asia-Pac J Clin Oncol..

[124] Elgart J, Caporale J, Gonzalez L, Aiello E, Waschbusch M, Gagliardino J (2013). Treatment of type 2 diabetes with saxagliptin: a pharmacoeconomic evaluation in Argentina. Health Econ Rev..

[125] Nita ME, Eliaschewitz FG, Ribeiro E, Asano E, Barbosa E, Takemoto M (2012). Cost-effectiveness and budget impact of saxagliptine as additional therapy to metformin for the treatment of diabetes mellitus type 2 in the Brazilian private health system. Rev Assoc Med Bras..

[126] Uruena A, Pippo T, Betelu MS, Virgilio F, Giglio N, Gentile A (2011). Cost-effectiveness analysis of the 10- and 13-valent pneumococcal conjugate vaccines in Argentina. Vaccine..

[127] Clark A, Jauregui B, Griffiths U, Janusz CB, Bolanos-Sierra B, Hajjeh R (2013). TRIVAC decision-support model for evaluating the cost-effectiveness of Haemophilus influenzae type b, pneumococcal and rotavirus vaccination. Vaccine..

[128] Demarteau N, Breuer T, Standaert B (2012). Selecting a mix of prevention strategies against cervical cancer for maximum efficiency with an optimization program. Pharmacoeconomics..

[129] Demarteau N, Morhason-Bello IO, Akinwunmi B, Adewole IF (2014). Modeling optimal cervical cancer prevention strategies in Nigeria. BMC Cancer..

[130] OpenMalaria, Swiss Tropical and Public Health Institute (Swiss TPH) and the Liverpool School of Tropical Medicine. 2015. https://github.com/SwissTPH/openmalaria/wiki. Accessed 22 June 2014.

[131] Steen R, Hontelez JAC, Veraart A, White RG, De Vlas SJ (2014). Looking upstream to prevent HIV transmission: Can interventions with sex workers alter the course of HIV epidemics in Africa as they did in Asia?. AIDS..

[132] van Vliet C, Meester EI, Korenromp EL, Singer B, Bakker R, Habbema JD (2001). Focusing strategies of condom use against HIV in different behavioural settings: an evaluation based on a simulation model. Bull World Health Organ..

[133] Stuckey EM, Stevenson JC, Cooke MK, Owaga C, Marube E, Oando G (2012). Simulation of malaria epidemiology and control in the highlands of Western Kenya. Malar J..

[134] Maskery B, Deroeck D, Levin A, Kim YE, Wierzba TF, Clemens JD (2013). Strategy, demand, management, and costs of an international cholera vaccine stockpile. J Infect Dis..

[135] Kim S-Y, Sweet S, Chang J, Goldie SJ (2011). Comparative evaluation of the potential impact of rotavirus versus HPV vaccination in GAVI-eligible countries: a preliminary analysis focused on the relative disease burden. BMC Infect Dis..

[136] Atashili J, Kalilani L, Seksaria V, Sickbert-Bennett EE (2008). Potential impact of infant feeding recommendations on mortality and HIV infection in children born to HIV-infected mothers in Africa: a simulation. BMC Infect Dis..

[137] Ciaranello AL, Seage GR, Freedberg KA, Weinstein MC, Lockman S, Walensky RP (2008). Antiretroviral drugs for preventing mother-to-child transmission of HIV in sub-Saharan Africa: balancing efficacy and infant toxicity. AIDS..

[138] Ciaranello AL, Perez F, Keatinge J, Park JE, Engelsmann B, Maruva M (2012). What will it take to eliminate pediatric HIV? Reaching WHO target rates of mother-to-child HIV transmission in Zimbabwe: a model-based analysis. PLoS Med..

[139] Alsallaq RA, Schiffer JT, Longini IM, Wald A, Corey L, Abu-Raddad LJ (2010). Population level impact of an imperfect prophylactic vaccine for herpes simplex virus-2. Sex Transm Dis..

[140] Amirfar S, Hollenberg JP, Abdool Karim SS (2006). Modeling the impact of a partially effective HIV vaccine on HIV infection and death among women and infants in South Africa. JAIDS..

[141] Maire N, Tediosi F, Ross A, Smith T (2006). Predictions of the epidemiologic impact of introducing a pre-erythrocytic vaccine into the expanded program on immunization in sub-Saharan Africa. Am J Trop Med Hyg..

[142] Stover J, Bollinger L, Hecht R, Williams C, Roca E (2007). The impact of an AIDS vaccine in developing countries: a new model and initial results. Health Aff..

[143] Gray RH, Li X, Wawer MJ, Gange SJ, Serwadda D, Sewankambo NK (2003). Stochastic simulation of the impact of antiretroviral therapy and HIV vaccines on HIV transmission. Rakai. Uganda AIDS..

[144] Andersson KM, Paltiel AD, Owens DK (2011). The potential impact of an HIV vaccine with rapidly waning protection on the epidemic in Southern Africa: examining the RV144 trial results. Vaccine..

[145] Andersson KM, Stover J (2011). The potential impact of a moderately effective HIV vaccine with rapidly waning protection in South Africa and Thailand. Vaccine..

[146] Andersson KM, Owens DK, Vardas E, Gray GE, McIntyre JA, Paltiel AD (2007). Predicting the impact of a partially effective HIV vaccine and subsequent risk behavior change on the heterosexual HIV epidemic in low- and middle-income countries: a South African example. J Acquir Immune Defic Syndr..

[147] Boccia TMQR, Burattini MN, Coutinho FAB, Massad E (2014). Will people change their vector-control practices in the presence of an imperfect dengue vaccine?. Epidemiol Infect..

[148] Bogard E, Kuntz KM (2002). The impact of a partially effective HIV vaccine on a population of intravenous drug users in Bangkok, Thailand: a dynamic model. J Acquir Immune Defic Syndr..

[149] Massad E, Coutinho FAB, Burattini MN, Lopez LF, Struchiner CJ (2001). Modeling the impact of imperfect HIV vaccines on the incidence of the infection. Math Comput Model..

[150] Bos JM, Postma MJ (2001). The economics of HIV vaccines: projecting the impact of HIV vaccination of infants in sub-Saharan Africa. Pharmacoeconomics..

[151] Lee BY, Bacon KM, Connor DL, Willig AM, Bailey RR (2010). The potential economic value of a Trypanosoma cruzi (Chagas disease) vaccine in Latin America. PLoS Negl Trop Dis..

[152] Lee BY, Bacon KM, Shah M, Kitchen SB, Connor DL, Slayton RB (2012). The economic value of a visceral leishmaniasis vaccine in Bihar state. India Am J Trop Med Hyg..

[153] Durham DP, Ndeffo Mbah ML, Medlock J, Luz PM, Meyers LA, Paltiel AD (2013). Dengue dynamics and vaccine cost-effectiveness in Brazil. Vaccine..

[154] Maire N, Shillcutt SD, Walker DG, Tediosi F, Smith TA (2011). Cost-effectiveness of the introduction of a pre-erythrocytic malaria vaccine into the expanded program on immunization in sub-Saharan Africa: analysis of uncertainties using a stochastic individual-based simulation model of Plasmodium falciparum malaria. Value Health..

[155] Tediosi F, Hutton G, Maire N, Smith TA, Ross A, Tanner M (2006). Predicting the cost-effectiveness of introducing a pre-erythrocytic malaria vaccine into the expanded program on immunization in Tanzania. Am J Trop Med Hyg..

[156] Leelahavarong P, Teerawattananon Y, Werayingyong P, Akaleephan C, Premsri N, Namwat C (2011). Is a HIV vaccine a viable option and at what price? An economic evaluation of adding HIV vaccination into existing prevention programs in Thailand. Public Health..

[157] Ono S, Kurotaki T, Nakasone T, Honda M, Boon-Long J, Sawanpanyalert P (2006). Cost-effectiveness analysis of antiretroviral drug treatment and HIV-1 vaccination in Thailand. Jpn J Infect Dis..

[158] Hecht R, Gandhi G (2008). Demand forecasting for preventive AIDS vaccines: economic and policy dimensions. Pharmacoeconomics..

[159] Azevedo RS, Amaku M (2011). Modelling immunization strategies with cytomegalovirus vaccine candidates. Epidemiol Infect..

[160] Hontelez JA, Nagelkerke N, Bärnighausen T, Bakker R, Tanser F, Newell ML (2011). The potential impact of RV144-like vaccines in rural South Africa: a study using the STDSIM microsimulation model. Vaccine..

[161] Schneider K, Kerr CC, Hoare A, Wilson DP (2011). Expected epidemiological impacts of introducing an HIV vaccine in Thailand: a model-based analysis. Vaccine..

[162] Nunes JK, Cárdenas V, Loucq C, Maire N, Smith T, Shaffer C (2013). Modeling the public health impact of malaria vaccines for developers and policymakers. BMC Infect Dis..

[163] Smith T, Killeen GF, Maire N, Ross A, Molineaux L, Tediosi F (2006). Mathematical modeling of the impact of malaria vaccines on the clinical epidemiology and natural history of Plasmodium falciparum malaria: overview. Am J Trop Med Hyg..

[164] Lee BY, Connor DL, Kitchen SB, Bacon KM, Shah M, Brown ST (2011). Economic value of dengue vaccine in Thailand. Am J Trop Med Hyg..

[165] Fonseca MGP, Forsythe S, Menezes A, Vuthoori S, Possas C, Veloso V (2010). Modeling HIV vaccines in Brazil: assessing the impact of a future HIV vaccine on reducing new infections, mortality and number of people receiving ARV. PLoS One..

[166] Brooks A, Briët OJT, Hardy D, Steketee R, Smith TA (2012). Simulated impact of RTS, S/AS01 vaccination programs in the context of changing malaria transmission. PLoS One..

[167] Johnson LF, Bekker LG, Dorrington RE (2007). HIV/AIDS vaccination in adolescents would be efficient and practical when vaccine supplies are limited. Vaccine..

[168] Smith T, Ross A, Maire N, Chitnis N, Studer A, Hardy D (2012). Ensemble modeling of the likely public health impact of a pre-erythrocytic malaria vaccine. PLoS Med..

[169] Lee BY, Bacon KM, Bailey R, Wiringa AE, Smith KJ (2011). The potential economic value of a hookworm vaccine. Vaccine..

[170] Nagelkerke NJ, Hontelez JA, De Vlas SJ (2011). The potential impact of an HIV vaccine with limited protection on HIV incidence in Thailand: a modeling study. Vaccine..

[171] Chao DL, Halstead SB, Halloran ME, Longini IM (2012). Controlling dengue with vaccines in Thailand. PLoS Negl Trop Dis..

[172] Massad E, Coutinho FA, Chaib E, Burattini MN (2009). Cost-effectiveness analysis of a hypothetical hepatitis C vaccine compared to antiviral therapy. Epidemiol Infect..

[173] Kaldor JM, Wilson DP (2010). How low can you go: the impact of a modestly effective HIV vaccine compared with male circumcision. AIDS..

[174] Seo MK, Baker P, Ngo KNL (2014). Cost-effectiveness analysis of vaccinating children in Malawi with RTS,S vaccines in comparison with long-lasting insecticide-treated nets. Malar J..

[175] Lee BY, Cakouros BE, Assi T-M, Connor DL, Welling J, Kone S (2012). The impact of making vaccines thermostable in Niger’s vaccine supply chain. Vaccine..

[176] Levin A, Levin C, Kristensen D, Matthias D (2007). An economic evaluation of thermostable vaccines in Cambodia. Ghana and Bangladesh. Vaccine..

[177] Alley WS, van Oortmarssen GGJ, Boatin BBA, Nagelkerke N, Plaisier AAP, Remme HJ (2001). Macrofilaricides and onchocerciasis control, mathematical modelling of the prospects for elimination. BMC Public Health..

[178] Dowdy DW, O’Brien MA, Bishai D (2008). Cost-effectiveness of novel diagnostic tools for the diagnosis of tuberculosis. Int J Tuberc Lung Dis..

[179] Dye C (2012). The potential impact of new diagnostic tests on tuberculosis epidemics. Indian J Med Res..

[180] Rheingans R, Atherly D, Anderson J (2012). Distributional impact of rotavirus vaccination in 25 GAVI countries: estimating disparities in benefits and cost-effectiveness. Vaccine..

[181] Killeen GF, Smith TA, Ferguson HM, Mshinda H, Abdulla S, Lengeler C (2007). Preventing childhood malaria in Africa by protecting adults from mosquitoes with insecticide-treated nets. PLoS Med..

[182] Cleary S, Mooney G, McIntyre D (2010). Equity and efficiency in HIV-treatment in South Africa: the contribution of mathematical programming to priority setting. Health Econ..

[183] Wilson DP, Blower SM (2005). Designing equitable antiretroviral allocation strategies in resource-constrained countries. PLoS Med..

[184] Moore B, Stamm JLH (2012). Impact of Decentralized Decision-Making on Access to Public Health Facilities. 62nd IIE Annual Conference and Expo 2012.

[185] Waters HR (2000). Measuring equity in access to health care. Soc Sci Med..

[186] McCoy JH, Lee HL (2014). Using fairness models to improve equity in health delivery fleet management. Prod Oper Manag..

[187] Guanqun L, Yamaki H, Sheng H. Mechanism Design Simulation for Healthcare Reform in China. In: Proceedings 12th International Conference on Principles of Practice in Multi-Agent Systems (PRIMA 2009). 2009. pp. 534–41. doi:10.1007/978-3-642-11161-7_39.

[188] Mathauer I, Musango L, Sibandze S, Mthethwa K, Carrin G (2011). Is universal coverage via social health insurance financially feasible in Swaziland?. S Afr Med J..

[189] McIntyre D, Ataguba JE (2012). Modelling the affordability and distributional implications of future health care financing options in South Africa. Health Policy Plan.

[190] Asfaw A, Von Braun J, Klasen S (2004). How big is the crowding-out effect of user fees in the rural areas of Ethiopia? Implications for equity and resources mobilization. World Dev..

[191] Sari N (2004). Consumer spending for pharmaceuticals and its implications for health care financing: The case of Kazakhstan. Eastern Eur Econ..

[192] Pagel C, Lewycka S, Colbourn T, Mwansambo C, Meguid T, Chiudzu G (2009). Estimation of potential effects of improved community-based drug provision, to augment health-facility strengthening, on maternal mortality due to post-partum haemorrhage and sepsis in sub-Saharan Africa: an equity-effectiveness model. Lancet..

[193] Carrera C, Azrack A, Begkoyian G, Pfaffmann J, Ribaira E, O’Connell T (2012). The comparative cost-effectiveness of an equity-focused approach to child survival, health, and nutrition: a modelling approach. Lancet..

[194] Kumar N (2004). Changing geographic access to and locational efficiency of health services in two Indian districts between 1981 and 1996. Soc Sci Med..

[195] Qingming Z, Xi W, Sliuzas R. A GIS-based Method to Assess the Shortage Areas of Community Health Service – Case Study in Wuhan, China. In: Proceedings of the 2011 International Conference on Remote Sensing, Environment and Transportation Engineering (RSETE 2011). IEEE; 2011. pp. 5654–7. doi:10.1109/RSETE.2011.5965635.

[196] Song J, Li B, Yang Y, Wu S, Griffin P. Improving Health Care Access by Optimizing the Allocation Network of Community Health Centers. In: 2008 IEEE International Conference on Service Operations, Logistics and Informatics (SOLI 2008). IEEE. 2008. pp. 933–8. doi:10.1109/SOLI.2008.4686532

[197] Alvis N, La Hoz DF, Gamboa O, Cediel N, Rico A, Paternina A (2011). Epidemiological and economic impact of tetanus vaccination in Colombian adults. Pan Am J Public Health..

[198] Andersson KM, Owens DK, Paltiel AD (2011). Scaling up circumcision programs in Southern Africa: The potential impact of gender disparities and changes in condom use behaviors on heterosexual HIV transmission. AIDS Behav..

[199] Dimitrov DT, Boily MC, Baggaley RF, Masse B (2011). Modeling the gender-specific impact of vaginal microbicides on HIV transmission. J Theor Biol..

[200] Dushoff J, Patocs A, Shi CF (2011). Modeling the population-level effects of male circumcision as an HIV-preventive measure: a gendered perspective. PLoS One..

[201] Kim JJ, Andres-Beck B, Goldie SJ (2007). The value of including boys in an HPV vaccination programme: a cost-effectiveness analysis in a low-resource setting. Brit J Cancer..

[202] Basu S, Babiarz KS, Ebrahim S, Vellakkal S, Stuckler D, Goldhaber-Fiebert JD (2013). Palm oil taxes and cardiovascular disease mortality in India: economic-epidemiologic model. BMJ..

[203] Basu S, Glantz S, Bitton A, Millett C (2013). The effect of tobacco control measures during a period of rising cardiovascular disease risk in India: a mathematical model of myocardial infarction and stroke. PLoS Med..

[204] Selvarajah S, Haniff J, Kaur G, Guat Hiong T, Bujang A, Chee Cheong K (2013). Identification of effective screening strategies for cardiovascular disease prevention in a developing country: using cardiovascular risk-estimation and risk-reduction tools for policy recommendations. BMC Cardiovasc Disord..

[205] Gomez GB, Borquez A, Caceres CF, Segura ER, Grant RM, Garnett GP (2012). The potential impact of pre-exposure prophylaxis for HIV prevention among men who have sex with men and transwomen in Lima. Peru: a mathematical modelling study. PLoS Med..

[206] Pretorius C, Stover J, Bollinger L, Bacaer N, Williams B (2010). Evaluating the cost-effectiveness of pre-exposure prophylaxis (PrEP) and its impact on HIV-1 transmission in South Africa. PLoS One..

[207] Wirtz AL, Pretorius C, Beyrer C, Baral S, Decker MR, Sherman SG (2014). Epidemic impacts of a community empowerment intervention for HIV prevention among female sex workers in generalized and concentrated epidemics. PLoS One..

[208] Adebayo SB, Fakolade R, Anyanti J, Ekweremadu B, Ladipo O, Ankomah A (2011). Modelling level, trend and geographical variations in stigma and discrimination against people living with HIV/AIDS in Nigeria. SAHARA J..

[209] Lasry A, Zaric GS, Carter MW (2007). Multi-level resource allocation for HIV prevention: a model for developing countries. Eur J Oper Res..

[210] Pretorius C, Menzies NA, Chindelevitch L, Cohen T, Cori A, Eaton JW (2014). The potential effects of changing HIV treatment policy on tuberculosis outcomes in South Africa: Results from three tuberculosis-HIV transmission models. AIDS..

[211] Walensky RP, Wood R, Ciaranello AL, Paltiel AD, Lorenzana SB, Anglaret X (2010). Scaling up the 2010 World Health Organization HIV treatment guidelines in resource-limited settings: a model-based analysis. PLoS Med..

[212] Walensky RP, Wood R, Fofana MO, Martinson NA, Losina E, April MD (2011). The clinical impact and cost-effectiveness of routine, voluntary HIV screening in South Africa. JAIDS..

[213] Dowdy DW, Steingart KR, Pai M (2011). Serological testing versus other strategies for diagnosis of active tuberculosis in India: a cost-effectiveness analysis. PLoS Med..

[214] Arita I, Nakane M, Fenner F (2006). Is polio eradication realistic?. Science..

[215] Thompson KM, Tebbens RJD (2008). Using system dynamics to develop policies that matter: global management of poliomyelitis and beyond. Syst Dynam Rev..

[216] Thompson KM, Tebbens RJ (2007). Eradication versus control for poliomyelitis: an economic analysis. Lancet..

[217] Roberts L (2007). Polio: no cheap way out. Science..

[218] The Global Polio Eradication Initiative. 2016. http://www.polioeradication.org. Accessed 4 Jan 2016.

[219] Hyder AA, Corluka A, Winch PJ, El-Shinnawy A, Ghassany H, Malekafzali H (2011). National policy-makers speak out: are researchers giving them what they need?. Health Policy Plan..

[220] Syed SB, Hyder AA, Bloom G, Sundaram S, Bhuiya A, Zhenzhong Z (2008). Exploring evidence-policy linkages in health research plans: a case study from six countries. Health Res Policy Syst..

[221] Mann G, Squire SB, Bissell K, Eliseev P (2010). Beyond accuracy: creating a comprehensive evidence base for TB diagnostic tools. Int J Tuberc Lung Dis..

[222] Okonkwo QL, Draisma G, der Kinderen A, Brown ML, de Koning HJ (2008). Breast cancer screening policies in developing countries: a cost-effectiveness analysis for India. J Natl Cancer Inst..

[223] Vanni T, Luz PM, Grinsztejn B, Veloso VG, Foss A, Mesa-Frias M (2011). Cervical cancer screening among HIV-infected women: an economic evaluation in a middle-income country. Int J Cancer..

[224] Evans T, Brown H (2003). Road traffic crashes: operationalizing equity in the context of health sector reform. Inj Control Saf Promot..

[225] O’Neill J, Tabish H, Welch V, Petticrew M, Pottie K, Clarke M (2014). Applying an equity lens to interventions: using PROGRESS ensures consideration of socially stratifying factors to illuminate inequities in health. J Clin Epidemiol..

[226] Oliver S, Kavanagh J, Caird J (2008). Health promotion, inequalities and young people’s health: a systematic review of research.

[227] Hennink M, Stephenson R (2005). Using research to inform health policy: barriers and strategies in developing countries. J Health Commun..

[228] Checchi F, Gayer M, Freeman Grais R, Mills EJ (2007). Public health in crisis-affected populations: a practical guide for decision-makers.

[229] Datta A, Pellini A (2011). Communicating Research: A Beginner’s Guide for Researchers in Vietnam.

[230] Hovland I (2005). Successful Communication: A Toolkit for Researchers and Civil Society Organizations.

[231] Bennett G, Jessani N, editors. The Knowledge Translation Toolkit - Bridging the Know-Do Gap: A Resource for Researchers. Ottawa: International Development Research Centre (IDRC); 2011.

[232] Haines A, Kuruvilla S, Borchert M (2004). Bridging the implementation gap between knowledge and action for health. Bull World Health Organ..

[233] Lewin T, Patterson Z (2012). Approaches to development research communication. IDS Bulletin..

[234] Hawkes S, Zaheer HA, Tawil O, O’Dwyer M, Buse K (2012). Managing research evidence to inform action: Influencing HIV policy to protect marginalised populations in Pakistan. Glob Public Health..

[235] World Bank (2016). Data: Countries and Economies.

