# Supplementary material for: Operations research in global health: a scoping review with a focus on the themes of health equity and impact
Source: Health Res Policy Syst. 2017 Apr 18;15:32. doi: 10.1186/s12961-017-0187-7 (PMC5395767; doi:10.1186/s12961-017-0187-7)
Supplement: Supplementary file 1 — Search strategies for Scopus, Compendex, Inspec and HealthStar databases. Description: Tables containing details of custom systematic search strategies for the Scopus, Compendex, Inspec and HealthStar databases. (DOCX 96 kb) [file 12961_2017_187_MOESM1_ESM.docx]

Additional file 1: Search strategies for Scopus, Compendex, Inspec and HealthStar databases.

Table S1: Example search strategy for Scopus database

| Database: **Scopus** |
| --- |
| Strategy: **Keyword search**. TITLE-ABS-KEY search, where KEY includes author keywords and controlled indexed terms in searched databases. Scopus automatically searches plural versions of words, as well as US-UK spelling variations. Each category combined with “AND”. Restricted to English and year 2000 or later. Irrelevant subject areas were excluded. |
| Sub-search categories:  ***(a) Model types***  TITLE-ABS-KEY=("operation* research") OR  TITLE-ABS-KEY =(model* W/5 (mathematical or queu* or inventory or scheduling or demand or forecast* or comput* or network or stochastic or decision* or delivery or simulation or optimization or non-linear or nonlinear or linear or Markov or cost-effectiveness or agent-based)) OR  TITLE-ABS-KEY =(optimization W/5 (mathematical or nonlinear or non-linear or linear or network or discrete or multicriteria or multi-criteria or stochastic or problem or minimization or maximization or location or allocation)) OR  TITLE-ABS-KEY =(simulation W/3 (comput* or discrete or agent-based or system$)) AND NOT  TITLE-ABS-KEY =("regression analysis" or “regression model”) |
| AND |
| ***(b) Geographic focus***  TITLE-ABS-KEY =("developing countr*" OR "low-income countr*" OR "middle-income countr*" OR "developing world" OR "developing nation*" OR "low-resource setting*" OR "resource-constrained setting*" OR "resource-poor setting*" OR "limited-resource setting*" OR "resource-limited setting*" OR "under-developed countr*" OR "least-developed countr*" OR "less-developed countr*" OR LMIC* OR Africa* OR (Asia* W/2 south) OR (Asia* W/2 east) OR "latin America*" OR "central America*" OR "south America*" OR Caribbean OR “middle east”) OR *[all low- and middle-income country names listed out]* |
| AND |
| ***(c) Health***  TITLE-ABS-KEY =(health* or medical or hospital or clinic* or treatment) OR AFFIL=(health) |
| AND |
| ***(d) Decision-/policy-making***  TITLE-ABS-KEY =(polic* or decision-mak* or decision-support or decision-process or decision-aid* or implement* or impact) OR AFFIL=(policy) |

Table S2: Example search strategy for Compendex database

| Database: **Compendex** |
| --- |
| Strategy: **Combination of controlled vocabulary (CV) and keyword (KY) search.** CV used for (a) model types, (c) health, and (d) decision-/policy-making terms. Searched KY in title/abs/subject for (b) geographic focus. Each category was combined with “AND”. Restricted to English and year 2000 or later. |
| Sub-search categories:  ***(a) Model types (used CV)***  Operations research; Mathematical techniques; Computational methods; Mathematical models; Optimization; Mathematical programming; Scheduling; Queueing networks; Queueing theory; Resource allocation; Random processes; Decision theory; Markov processes; Stochastic models; Computer simulation; Discrete event simulation; Linear programming; Inventory control; Dynamic programming; Cost effectiveness; Integer programming; Monte Carlo methods; Algorithms |
| AND |
| ***(b) Geographic focus (used KY)***  (developing NEAR/0 countr* OR low-income NEAR/0 countr* OR middle-income NEAR/0 countr* OR developing NEAR/0 world OR developing NEAR/0 nation* OR low-resource NEAR/0 setting* OR resource-constrained NEAR/0 setting* OR resource-poor NEAR/0 setting* OR limited-resource NEAR/0 setting* OR resource-limited NEAR/0 setting* OR under-developed NEAR/0 countr* OR least-developed NEAR/0 countr* OR less-developed NEAR/0 countr* OR LMIC* OR Africa* OR Asia* NEAR/2 south OR Asia* NEAR/2 east OR latin NEAR/0 America* OR central NEAR/0 America* OR south NEAR/0 America* OR caribbean OR middle NEAR/0 east WN KY) OR *[all low- and middle-income country names listed out]* |
| AND |
| ***(c) Health (used CV)***  Health; Health care; Hospitals; Home health care; Medicine; Public health; Patient treatment; Medical computing; Medical applications; Hospital data processing |
| AND |
| ***(d) Decision-/policy-making (used CV)***  Decision support systems; Decision making; Decision theory; Decision trees; Cost accounting; Public policy |

Table S3: Example search strategy for Inspec database

| Database: **Inspec** |
| --- |
| Strategy: **Combination of controlled vocabulary (CV) and keyword (KY) search.** CV used for (a) model types, (c) health, and (d) decision-/policy-making terms. Searched KY in title/abs/subject for (b) geographic focus. Each category was combined with “AND”. Restricted to English and year 2000 or later. |
| Sub-search categories:  ***(a) Model types (used CV)***  dynamic programming; linear programming; operations research; optimization; Monte Carlo methods; queueing theory; resource allocation; scheduling; simulation; stochastic processes; modeling; decision theory; integer programming; nonlinear programming; random processes; cost-benefit analysis; mathematical programming; Markov processes; discrete event simulation; digital simulation |
| AND |
| ***(b) Geographic focus (used KY)***  (developing NEAR/0 countr* OR low-income NEAR/0 countr* OR middle-income NEAR/0 countr* OR developing NEAR/0 world OR developing NEAR/0 nation* OR low-resource NEAR/0 setting* OR resource-constrained NEAR/0 setting* OR resource-poor NEAR/0 setting* OR limited-resource NEAR/0 setting* OR resource-limited NEAR/0 setting* OR under-developed NEAR/0 countr* OR least-developed NEAR/0 countr* OR less-developed NEAR/0 countr* OR LMIC* OR Africa* OR Asia* NEAR/2 south OR Asia* NEAR/2 east OR latin NEAR/0 America* OR central NEAR/0 America* OR south NEAR/0 America* OR caribbean OR middle NEAR/0 east WN KY) OR *[all low- and middle-income country names listed out]* |
| AND |
| ***(c) Health (used CV)***  Health care; Hospitals; Medicine; Patient care; Patient treatment; Medical computing; Medical administrative data processing |
| AND |
| ***(d) Decision-/policy-making (used CV)***  Decision support systems; Decision making; Decision theory; Decision trees |

Table S4: Example search strategy for HealthStar database

| Database: **HealthStar (via Ovid Medline)** |
| --- |
| Strategy: **Combined** **controlled vocabulary search using MeSH subject headings and custom keyword search.** Since HealthStar contains citations to published literature on health services, technology, administration, and research, search terms were not developed for the health or decision-/policy-making categories. MeSH and keyword search terms selected for model type and geographic focus only. Each category was combined with “AND”. Restricted to English, year 2000 or later, and relevant document types. |
| Sub-search categories:  ***(a) Model types***  *MeSH subject headings*  decision support techniques; models, economic; models, econometric; Monte Carlo method; Markov chains; stochastic processes; algorithms; decision theory; decision trees; operations research; systems theory; computing methodologies; computer simulation; mathematical computing; numerical analysis, computer-assisted; systems analysis; probability theory; programming, linear; cost-benefit analysis; health services research; decision-making, organizational; combined with the following custom search in *titles* and *keywords:*  *Custom search*  (operation* adj1 research) OR  (model* adj5 mathematical or queu* or inventory or scheduling or demand or forecast* or comput* or network or stochastic or decision* or delivery or simulation or optimi#ation or linear or nonlinear or non-linear or Markov or cost-effectiveness or agent-based) OR  (optimi#ation$1 adj5 mathematical or nonlinear or non-linear or linear or network or discrete or multi-criteria or multicriteria or stochastic or problem or minimi#ation or maximi#ation or location or allocation) OR  (simulation adj3 comput* or discrete or agent-based or system$1) |
| AND |
| ***(b) Geographic focus***  *MeSH subject headings*  Selected all low- and middle-income countries (according to World Bank classifications) in Geographicals (MeSH subject headings) for each continent; combined with the following custom search in *titles* and *keywords*:  *Custom search*  (developing countr* or low-income countr* or middle-income countr* or developing world or developing nation* or low-resource setting* or resource-constrained setting* or resource-poor setting* or limited-resource setting* or resource-limited setting* or under-developed countr* or least-developed countr* or less-developed countr* or LMIC*) |
